# Characterizing phenotypic diversity of trehalose biosynthesis mutants in multiple wild strains of *Saccharomyces cerevisiae*

**DOI:** 10.1093/g3journal/jkac196

**Published:** 2022-08-05

**Authors:** Anqi Chen, Jeremy R Smith, Hugo Tapia, Patrick A Gibney

**Affiliations:** Department of Food Science, Cornell University, Ithaca, NY 14853, USA; Department of Food Science, Cornell University, Ithaca, NY 14853, USA; Biology Program, California State University—Channel Islands, Camarillo, CA 93012, USA; Department of Food Science, Cornell University, Ithaca, NY 14853, USA

**Keywords:** trehalose biosynthesis, *Saccharomyces cerevisiae*, *tps1* catalytically inactive alleles, genetic heterogeneity, *TPS1*, *TPS2*, *TPS3*, *TSL1*

## Abstract

In the yeast *Saccharomyces cerevisiae*, trehalose-6-phospahte synthase (Tps1) and trehalose-6-phosphate phosphatase (Tps2) are the main proteins catalyzing intracellular trehalose production. In addition to Tps1 and Tps2, 2 putative regulatory proteins with less clearly defined roles also appear to be involved with trehalose production, Tps3 and Tsl1. While this pathway has been extensively studied in laboratory strains of *S. cerevisiae*, we sought to examine the phenotypic consequences of disrupting these genes in wild strains. Here we deleted the *TPS1*, *TPS2*, *TPS3*, and *TSL1* genes in 4 wild strains and 1 laboratory strain for comparison. Although some tested phenotypes were not shared between all strains, deletion of *TPS1* abolished intracellular trehalose, caused inability to grow on fermentable carbon sources and resulted in severe sporulation deficiency for all 5 strains. After examining *tps1* mutant strains expressing catalytically inactive variants of Tps1, our results indicate that Tps1, independent of trehalose production, is a key component for yeast survival in response to heat stress, for regulating sporulation, and growth on fermentable sugars. All *tps2*Δ mutants exhibited growth impairment on nonfermentable carbon sources, whereas variations were observed in trehalose synthesis, thermosensitivity and sporulation efficiency. *tps3*Δ and *tsl1*Δ mutants exhibited mild or no phenotypic disparity from their isogenic wild type although double mutants *tps3*Δ *tsl1*Δ decreased the amount of intracellular trehalose production in all 5 strains by 17–45%. Altogether, we evaluated, confirmed, and expanded the phenotypic characteristics associated trehalose biosynthesis mutants. We also identified natural phenotypic variants in multiple strains that could be used to genetically dissect the basis of these traits and then develop mechanistic models connecting trehalose metabolism to diverse cellular processes.

## Introduction

*Saccharomyces cerevisiae* is one of the most extensively characterized eukaryotes (Botstein and Fink 2011; Duina *et al.* 2014). However, research on *S. cerevisiae* has historically focused on a limited number of strains, or genetic backgrounds. In particular, derivatives of the strain S288C are some of the most commonly used laboratory strains, including the derivative used to produce the first completely sequenced, assembled, and annotated eukaryotic genome (Mortimer and Johnston 1986; Goffeau *et al.* 1996; Brachmann *et al.* 1998; Engel *et al.* 2014). These laboratory strains have also been used as powerful tools for functional genome analyses (Botstein and Fink 2011; Giaever and Nislow 2014). However, there are many different *S. cerevisiae* strains, and much of the phenotypic variation between strains remains unexplored. Extensive analysis has been performed to examine the nucleotide sequence diversity between *S. cerevisiae* strains from diverse backgrounds and found that the genomes of wild and laboratory strains have significant genetic variability (Dunn *et al.* 2005; Liti *et al.* 2009; Borneman *et al.* 2016; Gallone *et al.* 2016; Peter *et al.* 2018). While laboratory strains of *S. cerevisiae* have been, and continue to be, useful tools for understanding fundamental aspects of eukaryotic biology, comparative genetic analysis between wild and lab strains, or simply examining wild strains, can provide further biological insight (Ruderfer *et al.* 2006; Botstein and Fink 2011; Hittinger 2013; Peter *et al.* 2018). For example, the list of “essential” genes in *S. cerevisiae* varies somewhat from strain to strain (Chin *et al.* 2012). Strain-specific phenotypic variations can provide insight into the prevalence of selected phenotypes across strains and provide tools for characterizing the genetic basis of observed phenotypic variance. Trehalose metabolism is an example of a biological process in yeast that has been thoroughly characterized in lab strains; however, an in-depth analysis of phenotypic variations in wild yeasts has yet to be performed (Van Aelst *et al.* 1993; Gibney *et al.* 2015, 2020).

**Fig. 1.**
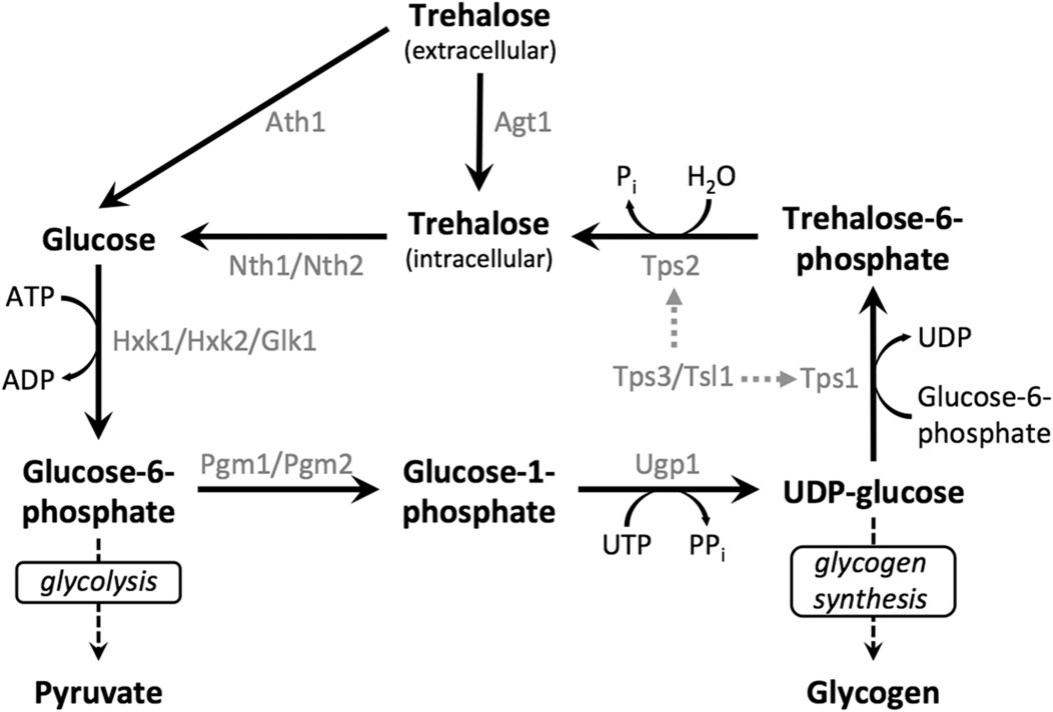
Schematic of trehalose metabolism in *S. cerevisiae*. Main pathway metabolites are shown in bolded text. Enzymes are shown in gray text. Dashed arrows associated with Tps3/Tsl1 indicate unknown mechanisms associated with supporting Tps1 and Tps2 function. To provide metabolic context, the glycolysis and glycogen synthesis pathways are also indicated.

Trehalose is a nonreducing disaccharide of α-(1,1)-linked glucose present in many organisms, including bacteria, fungi, insects, and plants (Elbein *et al.* 2003). In the yeast *S. cerevisiae*, trehalose synthesis involves 2 main enzymes: trehalose-6-phosphate synthase (Tps1), which catalyzes the synthesis of trehalose-6-phosphate (T6P), and trehalose-6-phosphate phosphatase (Tps2), which dephosphorylates T6P to trehalose (Fig. 1). The trehalose synthesis complex (TPS) in yeast includes 2 other proteins, Tps3 and Tsl1, which are 2 putative regulatory subunits (Reinders *et al.* 1997; Bell *et al.* 1998). The TPS complex was thought to include Tps1 (56 kDa), Tps2 (102 kDa), and Tsl1 (123k Da), whereas Tps3 (118 kDa) is a paralog of Tsl1 remaining from an ancestral whole genome duplication in the *S. cerevisiae* lineage and was discovered later (Panek *et al.* 1987; Vandercammen *et al.* 1989; Londesborough and Vuorio 1993; de Mesquita *et al.* 2003; Kellis *et al.* 2003; Voit 2003; Byrne and Wolfe 2005; Mahmud *et al.* 2010; Trevisol *et al.* 2014). The yeast 2-hybrid approach revealed that there is no interaction between Tps3 and Tsl1, but both interact with Tps1 and Tps2 which, in turn, interact with each other (Reinders *et al.* 1997). These observations, along with cofractionation experiments for detecting physical association of proteins, suggest a model where the TPS complex may be stabilized by protein–protein interactions with either Tsl1 or Tps3, though comprehensive testing of this model remains to be performed. Interestingly, all 4 protein components of the TPS complex have blocks of amino acid homology, potentially suggesting a common ancestor (de Virgilio *et al.* 1993; Vuorio *et al.* 1993).

Mutants of trehalose biosynthesis genes in *S. cerevisiae* have been characterized in many previous studies in multiple laboratory strains, including S288C derivatives, and diverse phenotypes have been observed (Gancedo and Flores 2004). For example, *tps1* mutants not only lack metabolic ability to synthesize T6P or trehalose but also exhibit several pleiotropic defects including inability to utilize fermentable carbon sources, thermosensitivity, alterations in glycogen levels, and sporulation deficiency (Hottiger *et al.* 1989; González *et al.* 1992; Hohmann *et al.* 1993, 1996; Van Aelst *et al.* 1993; Argüelles 1994; Neves *et al.* 1995; Singer and Lindquist 1998a; Enyenihi and Saunders 2003; Gancedo and Flores 2004; Shi *et al.* 2010; Van Heerden *et al.* 2014; Gibney *et al.* 2015; Liu *et al.* 2020). While several hypotheses and metabolic models have been proposed, the molecular mechanism underlying these unrelated phenotypes associated with this mutant remains elusive. A number of observations suggest that components of the trehalose metabolism pathway may have other functions beyond production of trehalose. For example, in the rice blast fungus *Magnaporthe grisiae*, Tps1 possesses an independent regulatory function essential for plant infection by the fungus, in addition to T6P synthase enzymatic activity (Wilson *et al.* 2007). Additionally, in *S. cerevisiae*, accumulated intracellular trehalose failed to repair many of the phenotypes associated with *tps1*, suggesting that absence of this disaccharide does not cause these phenotypes (Ratnakumar and Tunnacliffe 2006; Calahan *et al.* 2011; Gibney *et al.* 2015). Other components in the TPS complex also exhibit diverse phenotypes: similar to *tps1* mutants, *tps2* mutants are also heat sensitive and sporulation defective, but often exhibit growth defects on non-fermentable sugars (Piper and Lockheart 1988; de Virgilio *et al.* 1993; Thevelein and Hohmann 1995; Elliott *et al.* 1996; Enyenihi and Saunders 2003; Gibney *et al.* 2015). The other 2 proteins involved in trehalose biosynthesis, Tps3 and Tsl1, have been the least studied. In the W303 genetic background, both single mutants of *TPS3* or *TSL1* decreased activity of Tps1 and Tps2, while trehalose levels were reduced to approximately 50% in the *tps3 tsl1* double mutant (Bell *et al.* 1998).

To evaluate phenotypic diversity of trehalose biosynthesis mutants in wild strains compared to lab strains, we deleted each of the 4 biosynthetic genes in 5 different strains. We included 2 commercial wine strains (Simi White and CSM), 1 vineyard isolate (Bb32(3)), 1 oak-tree isolate (YPS1000), and an S288C derivative for comparison. After examining multiple phenotypes for these mutants, we were able to identify common phenotypes shared amongst most strains, in addition to strain-specific variations that could be used for future genetic analysis. To further investigate the pleiotropic phenotypes associated with *tps1*Δ, we also examined a number of mutant *TPS1* alleles that produce protein variants unable to synthesize T6P. Using these alleles, we provide further evidence that a number of the phenotypes associated with trehalose metabolism are not directly related to intracellular trehalose levels. This strengthens the notion that some other aspect of trehalose metabolism has an independent function. Together these results provide a comparison of trehalose biosynthesis mutant phenotypes from diverse strains, which was used to both confirm and expand our understanding of how trehalose metabolism is integrated into the cellular growth, stress response, and signaling network.

## Materials and methods

### Yeast growth media

Yeast cell growth and standard laboratory manipulations were performed using standard approaches (Botstein and Fink 2011). All media used were either minimal (YNB: 0.67% w/v yeast nitrogen base without amino acids plus 2% w/v indicated carbon sources), or rich (YP: 2% w/v Bacto peptone, 1% w/v yeast extract, 2% w/v indicated carbon sources). Solid media formulations included 2% w/v agar and were poured into standard 10 cm plastic Petri dishes or rectangular plates (Petri 1887). Exceptions are YPGE and SGE media, rich and minimal formulations, respectively, containing 3% v/v glycerol and 2% v/v ethanol as respiratory carbon sources.

### Yeast growth

Measurements of cell density were performed by measuring absorbance at 600 nm using a Gensys 6 UV-Vis spectrophotometer (Thermo Fisher). Measurements of cell density and cell size were performed using a Coulter Z2 Particle Count and Size Analyzer (Beckman Coulter) with a 100-μm aperature. For comparative growth assays, cells were spotted onto relevant solid growth media. Cell spotting was performed by dilution of a stationary phase culture to an initial OD_600_ of 1.0, followed by 10-fold serial dilutions. All dilutions were then spotted onto solid media using a Replica Plater for 96-well Plate, either the 8 × 6 or 12 × 8 array as needed (Sigma-Aldrich). Plates were incubated at indicated temperatures for indicated times as noted in the figures and legends. At least 3 independent biological replicates were performed on different days for spotting assays shown in figures, and a representative image is shown.

### Yeast strain construction

The strains used in this study are listed in Supplementary Table 1. Gene deletions were constructed by transforming PCR products amplified from plasmids containing different deletion cassettes: pFA6a-kanMX for kanMX, pAG32 for hphMX, and pAC372 for natAC (Supplementary Table 2). Primers were designed with 40 flanking base pairs identical to the upstream and downstream region of genes to be deleted by homologous recombination. All gene deletions in the S288C background were made by transformation into a diploid to get a heterozygote, which was confirmed by PCR then dissected to get MAT**a** and MATα segregants before mating together to obtain a homozygous diploid, which was also confirmed by PCR. Similarly, mutant strains constructed from non-S288C strains (Simi White, YPS1000, CSM, and Bb32) were made in the same way although homozygous diploids were obtained directly after dissection (the wild strains are homothallic; spores germinate, then cells switch mating types, and mate with each other to produce colonies that are essentially all diploid cells). All combinatorial gene deletion/insertion strains were made by mating, sporulating, and tetrad dissection. Sporulation was performed by growing cells to log phase in rich media, collecting cells by centrifugation, washing once in 1% w/v potassium acetate, and then resuspending in 1% w/v potassium acetate. Cells were then incubated at room temperature on a roller wheel for at least 6 days before tetrad dissection.

### Plasmid construction

Plasmids were built using pRS-series shuttle vector backbones containing the *THD3* promoter and *CYC1* 3′ UTR (Sikorski and Hieter 1989; Mumberg *et al.* 1995). All inserts were amplified using primers from Integrated DNA Technologies (IDT), and final PCR products contained a 5′-end SpeI and 3′-end XhoI sites to use for restriction enzyme-based confirmation of inserts during cloning (Supplementary Table 2). To construct plasmids containing different *tps1* variants (Tps1^R24V^, Tps1^Y102V^, Tps1^W111S^, Tps1^D156G^, and Tps1^Y102V, W111S^) and *otsA*, linearized p416GPD (using SpeI and XhoI) was transformed into *ura3Δ0* yeast cells along with different PCR inserts for each gene/allele containing 40 bp of flanking sequence identical to the 3′ end of the *TDH3* promoter or to the 5′ end of the *CYC1* 3′ UTR present in p416GPD. Plasmids assembled by homologous recombination in yeast after transforming both fragments were then extracted using Zymoprep Yeast Plasmid MiniPrep II kit (Zymo Research). Extracted plasmids were transformed into TOP10 *E. coli* for storage and insert confirmation. During construction of these mutant-allele-containing plasmids, we inadvertently also constructed an allele containing 2 amino acid substitutions (Y102V and W111S). As this allele would also be predicted to be nonfunctional for T6P synthesis, we decided to include it for further analysis. *MKT1* expression plasmids were cloned using Gibson Assembly (Supplementary Table 2) (Gibson *et al.* 2009). Linearized p426GPD (using SpeI and XhoI) was incubated with *MKT1* alleles amplified from their respective strain backgrounds along with the Gibson Assembly mixture at 50°C for 1 h before transforming into TOP10 *E. coli*. After transformation, individual colonies were screened for correct insertion using restriction digest. All gene insertion and allele-specific mutations were confirmed by Sanger Sequencing at the Cornell Institute of Biotechnology sequencing core facility.

### Assessment of thermotolerance

To assess thermotolerance, minimal media cultures were inoculated with a single colony and grown overnight. Cells were then diluted into the same minimal medium to an OD_600_ = 0.05 and grown another 24 h to stationary phase. Two aliquots of 0.8 ml cell culture were removed into microcentrifuge tubes. For the heat shock, one of the aliquots was incubated in a 42°C thermomixer for 2 h. Both pre- and post-heat shocked cell dilutions were plated on rich media (YPD or YPGal, depending on strain growth requirements) and incubated at 30°C for 2–3 days to measure viability by counting colony forming units. At least 3 independent biological replicates were performed for each thermotolerance assay.

### Measurement of sporulation efficiency

Sporulation was performed by growing cells to log phase in rich media (except cells containing plasmid-based *tps1* catalytically inactive alleles which were grown in minimal media), collecting cells by centrifugation, washing twice in 1% w/v potassium acetate, then resuspending in 1% w/v potassium acetate. Cells were then incubated at room temperature on a roller wheel for at least 6 days before calculating % sporulation by counting at least 300 cells. Sporulation efficiency was calculated as the proportion of observed tetrads compared to the total number of observed cells. At least 3 independent biological replicates were performed for each sporulation efficiency assay.

### Measurement of trehalose and glycogen

Trehalose and glycogen levels were measured essentially as described (Parrou and François 1997). Briefly, 10 OD_600_ units of stationary phase cells were isolated, washed in cold water, and resuspended in 250 μl of 0.25 M sodium carbonate. Cell mixtures were then stored at −80°C until ready to perform the assay. To begin the assay, cells were boiled at 95°C for 4 h with occasional agitation—this step extracts the trehalose and glycogen, as both are highly stable and not degraded. Next, 150 μl of acetic acid was added to the sample, followed by 600 μl of 0.2 M sodium acetate. After mixing, 350 μl was removed to a fresh tube, and 5 μl of 70 U/ml trehalase (Megazyme) or 70 U/ml amyloglucosidase (Sigma-Aldrich) was added. This was incubated overnight at 37°C or 57°C, respectively, in a thermomixer set at 550 rpm (Eppendorf). Next, the sample was centrifuged at maximum speed for 3 min, and 200 μl of each sample was used to measure the amount of glucose liberated from trehalose or glycogen using the Glucose (GO) Assay Kit (Sigma Aldrich).

### Statistical analysis

All experiments were conducted using at least 3 independent biological replicates. Mutants were evaluated for statistical significance compared to their isogenic wild type strains using a paired *t* test and are presented as the mean and standard deviation. The asterisks (*) indicate the mutant phenotype showed a difference (*P* < 0.05) compared to its isogenic wild type (*P*-values were not corrected for multiple hypothesis testing). Separately, to evaluate statistically significant phenotypic differences between the wild type strains examined, or also between strains with identical gene deletions, 1-way ANOVA with post-hoc Tukey HSD tests were performed (Supplementary Tables 3–8). Indicated *P*-values corresponding to the *F*-statistic of 1-way ANOVA lower than 0.05, suggest that the one or more evaluated treatments are significantly different.

## Results and discussion

### Trehalose mutant phenotypes under consideration

Multiple phenotypes have been ascribed to different trehalose mutants. To systematically examine the phenotypic diversity of each trehalose biosynthesis gene deletion mutant (*TPS1*, *TPS2*, *TPS3*, *TSL1*) and 1 double deletion mutant (*TPS3* and *TSL1*), we deleted each gene in 5 different strains of *S. cerevisiae*. These strains included 2 commercial wine strains (Simi White and CSM), 1 vineyard isolate (Bb32(3)), 1 oak-tree isolate (YPS1000), and an S288C derivative for comparison. Regarding phenotypes, we opted to focus on a core set of relevant phenotypes that could be assayed for each mutant. Because trehalose mutants have variations in both trehalose and glycogen levels, we quantified both. Further, stress sensitivity and sporulation defects are common phenotypes for *tps1*Δ and *tps2*Δ deletion mutants, so we assessed thermotolerance (42°C for 2 h), ability to grow at an elevated temperature (growth at 37°C), and ability to perform meiosis/sporulation. We also assessed cell size and growth on multiple carbon sources in both rich and minimal medium.

### Construction and phenotypic characterization of *tps1*Δ

*TPS1* encodes the trehalose-6-phosphate synthetase enzyme, which catalyzes the joining of glucose-6-phosphate with the glycosyl unit from UDP-glucose (Fig. 1). Deletion of the *TPS1* gene is associated with a variety of growth phenotypes, including failure to grow on fermentable carbon sources (e.g. glucose, fructose), failure to grow at elevated temperatures, failure to sporulate, and higher levels of glycogen accumulation (Gancedo and Flores 2004). Variations in some of these phenotypes, such as carbon source utilization, have been reported previously. For example, while a small subpopulation of S288C *tps1*Δ cells have the ability to grow on glucose at roughly 1 in 1,000, deleting *TPS1* in W303 genetic background almost completely abolishes their growth on the same sugar (Van Heerden *et al.* 2014; Gibney *et al.* 2020). Elucidating the genetic underpinnings regulating such differences will have a significant impact in our understanding of the tested cellular processes.

**Fig. 2.**
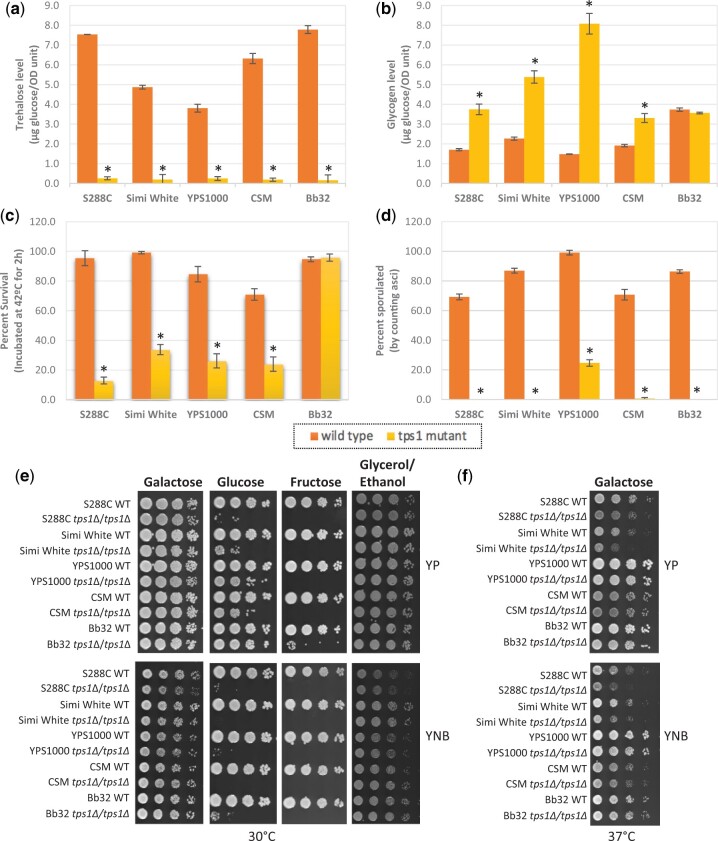
*tps1*Δ mutant phenotypes. a) Intracellular trehalose, b) intracellular glycogen, c) thermotolerance, d) sporulation efficiency, e) growth at 30°C for 2 days, and f) growth at 37°C for 3 days. For panels (e) and (f), indicated strains were grown overnight in YNB + 2% galactose liquid before performing assays as described in *Materials and Methods*. For plate images, 10-fold serial dilutions were prepared and spotted onto the indicated media. The initial dilution had an OD_600_ of 1.0. Three biological replicates were performed for all tested phenotypes. Asterisks represent statistical difference (*P* < 0.05) between the mutants and their isogenic wild types. Strain legend below (c) and (d) applied to (a)–(d).

Trehalose and glycogen levels were both determined using stationary phase cells because the cellular content of both carbohydrates is barely detectable in exponential phase even in wild type cells (Yi *et al.* 2016). As expected, no trehalose was produced in all 5 *tps1*Δ strains whereas the wild type strains showed a variety of intracellular trehalose levels (Fig. 2a, Supplementary Table 4). On the other hand, previous work demonstrated that glycogen levels are increased compared to wild type in S288C, CEN.PK, and other lab *tps1*Δ strains (Cannon *et al.* 1994; Guillou *et al.* 2004; Shi *et al.* 2010). One potential explanation is that absence of Tps1 results in excess UDP-glucose being shunted to glycogen production (Fig. 1) (Shi *et al.* 2010). While most *tps1*Δ strains did have significantly higher glycogen levels than wild type, Bb32 *tps1*Δ was notable in that glycogen levels for this strain remained identical to wild type cells (Fig. 2b), demonstrating that trehalose and glycogen levels are not necessarily negatively correlated. While characterizing these strains, we also noted a slight increase in cell size in Simi White *tps1*Δ and CSM *tps1*Δ, 4% and 6% larger, respectively, though no significant differences in cell size were detected in the other strain backgrounds (Supplementary Fig. 2a).

**Fig. 3.**
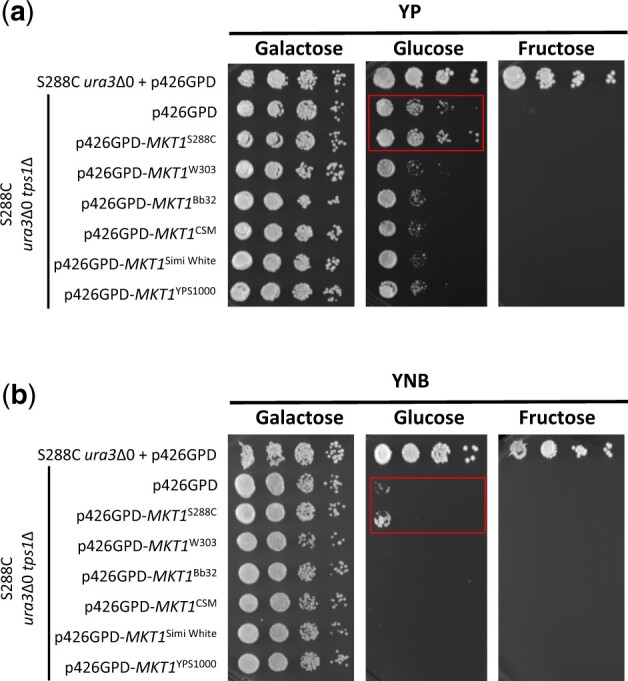
S288C *tps1*Δ persister-like cells can be prevented by overexpressing *MKT1* allele from different genetic backgrounds. Indicated strains were grown overnight in YNB + 2% galactose liquid before 10-fold serial dilutions were prepared and spotted onto the indicated media. The initial dilution had an OD_600_ of 1.0. Listed carbon sources were present at 2%. Plates were incubated at 30°C for 2 days on (a) rich media and 3 days for (b) minimal media. Red boxes highlight the *tps1*Δ persister-like cell colonies.

Heat sensitivity of lab *tps1*Δ strains has been reported in a number of previous studies (Hottiger *et al.* 1989; Eleutherio *et al.* 1993; Argüelles 1994; Singer and Lindquist 1998b; Gibney *et al.* 2015). Thermotolerance refers to the ability of cells to survive when treated with a lethal heat dosage. Here, thermotolerance of *tps1*Δ mutants was assessed by incubating stationary phase cells at 42°C for 2 h. In response to this heat shock, the viability of most *tps1*Δ mutants dropped significantly. Interestingly, Bb32 *tps1*Δ did not exhibit heat sensitivity and maintained wild type levels of viability (Fig. 2c). Trehalose has been proposed to protect denatured proteins from aggregation and therefore protect cells against thermal stress (Singer and Lindquist 1998a; Jain and Roy 2009). This model for trehalose function is based on a correlation of trehalose production during cellular stress along with direct observation of trehalose-mediated prevention of denatured protein aggregation *in vitro* (Singer and Lindquist 1998a). However, a direct test of intracellular trehalose as a thermoprotectant indicated that accumulation of this disaccharide inside cells did not repair *tps1*Δ thermotolerance defect (Gibney *et al.* 2015). Similarly, Bb32 *tps1*Δ cells do not produce trehalose but have wild type levels of thermotolerance, providing further evidence that high intracellular trehalose levels are not required for heat resistance. To further understand this particular phenotype, Bb32 *tps1*Δ could be used as a tool for future work to dissect the genetic underpinnings of *tps1*Δ thermosensitivity. In addition to examining acute thermotolerance at a temperature too high for long-term cellular survival, we also examined the ability of these mutants to grow at 37°C—a milder heat stress that each wild type strain can adapt to and grow (Fig. 2f andSupplementary Fig. 3). The viability and/or growth rate of S288C *tps1*Δ and Simi White *tps1*Δ were noticeably compromised compared to their wild types, though the *tps1*Δ mutants in the remaining 3 strains did not exhibit an obvious temperature-sensitive growth phenotype.

Homozygous *TPS1* deletion mutants are unable to sporulate in S288C-derived and W303 laboratory strains (Van Aelst *et al.* 1993; de Silva-Udawatta and Cannon 2001; Enyenihi and Saunders 2003; Gibney *et al.* 2015; Liu *et al.* 2020). However, this is not a shared phenotype to all laboratory strains as in the SK1 genetic background, we observed that 60% of *tps1*Δ mutant cells are still able to sporulate, suggesting that the sporulation defect associated with this mutant may be specific to certain lab strains (Supplementary Fig. 4). In contrast, all 5 tested *tps1*Δ strains exhibited sporulation defects compared to their isogenic wild type strains which had sporulation efficiencies between 60% and 100% (Fig. 2d). Four out of 5 did not sporulate at all (Fig. 2d). Roughly 20% of YPS1000 *tps1*Δ cells were able to sporulate, though this was still significantly lower than the YPS1000 wild type strain (Fig. 2d). One hypothesis to explain this defect is the absence of trehalose. However, Gibney *et al.* demonstrated that the sporulation defect cannot be fixed by adding intracellular trehalose to an S288C *tps1*Δ before sporulation (Gibney *et al.* 2015). The mechanistic basis connecting trehalose metabolism to sporulation remains unexplained, though overexpression of either *IME1* or *IME2*, transcription factors that regulate meiosis, is able to suppress the *tps1*Δ sporulation defect (de Silva-Udawatta and Cannon 2001). As with thermosensitivity, strain-to-strain variation in this phenotype could be a useful tool for future study to dissect the mechanistic basis of this phenotype.

The most widely studied phenotype of *tps1*Δ is its inability to grow on rapidly fermentable carbon sources such as glucose and fructose (Navon *et al.* 1979; González *et al.* 1992; Stucka and Blázquez 1993; Van Aelst *et al.* 1993; Hohmann *et al.* 1994, 1996; Luyten *et al.* 1995; Neves *et al.* 1995). Two main models have been historically proposed to explain this deficiency (Thevelein and Hohmann 1995; Walther *et al.* 2013). First, trehalose-6-phosphate (T6P) slows down glycolysis by inhibiting Hxk2, the major fermentative hexokinase enzyme, and that without Tps1 or T6P, metabolic flux through upper glycolysis consumes more ATP than lower glycolysis can produce, causing cessation of growth (Blázquez *et al.* 1993; Hohmann *et al.* 1996). Second, the trehalose pathway is important for releasing inorganic phosphate during fermentation that can be used as a substrate for glyceraldehyde-3-phosphate dehydrogenase in lower glycolysis (Van Aelst *et al.* 1993; Van Heerden *et al.* 2014). However, contrary experimental evidence demonstrates that neither of these models can fully explain the observed phenotypes. For example, T6P inhibition of Hxk2 has been demonstrated *in vitro*, but the *in vivo* relevance has not been directly examined (Hohmann *et al.* 1993). Further, overexpression of a T6P-insensitive hexokinase enzyme from *Schizosaccharomyces pombe* in *S. cerevisiae* did not affect glucose growth and only had minor effects on the short-term response to glucose exposure (Bonini *et al.* 2003). Regarding phosphate release for lower glycolysis, *tps2*Δ mutants also accumulate phosphate in T6P, yet are able to grow well on glucose and fructose (Van Heerden *et al.* 2014; Gibney *et al.* 2015). More recent work has implicated persistent decreased intracellular pH associated with glucose-exposed *tps1*Δ cells as at least partially responsible for the metabolic block at glyceraldehyde-3-phosphate dehydrogenase (van Leemputte *et al.* 2020). Another recent proposal to explain this fermentable carbon source growth defect is based on the observation that glucose-exposed *tps1*Δ mutants hyperaccumulate fructose-1,6-bisphosphate, which subsequently activates Ras and triggers apoptosis (Peeters *et al.* 2017). Taken together, these models and some of the differences between each model indicate a need to develop a more comprehensive model for the phenotypic consequences of trehalose metabolism that can explain observed phenotypes. When testing carbon source utilization, all 5 *tps1*Δ strains grew well on media containing respiratory carbon sources galactose (preferred respiration) or glycerol/ethanol (obligate respiration) (Fig. 2e). None of the *tps1*Δ strains grew on fructose, as observed with most laboratory strains, though notably Bb32 *tps1*Δ exhibited a small number of cells able to grow in rich, fructose-containing medium. Interestingly, all 5 *tps1*Δ strains showed varying levels of growth on rich glucose medium, and a few strains showed slight growth on minimal glucose medium (Fig. 2e). We were somewhat surprised by the varying levels of *tps1*Δ growth observed on glucose. Multiple laboratory strains are completely unable to grow on glucose in rich or minimal media (Van Aelst *et al.* 1993; Vuorio *et al.* 1993). In contrast, we and others recently demonstrated a subpopulation of S288C *tps1*Δ mutant cells able to grow on glucose, that this phenotype is not stably genetically propagated, is enhanced by peptone, and does not confer the ability to grow on fructose (Van Heerden *et al.* 2014; Gibney *et al.* 2020). We termed these persister-like cells and demonstrated that robust S288C *tps1*Δ persister-like cell formation requires a loss-of-function *MKT1* allele common to the S288C background (Gibney *et al.* 2020). To characterize glucose growth in these wild *tps1*Δ strains, we tested whether glucose growth was stably, genetically propagated to all daughter cells and found persister-like cell behavior instead (Supplementary Fig. 5). Further, similar to persister-like cells, this phenotype is enhanced in rich media and absent in fructose-containing media (Fig. 2e andSupplementary Fig. 5). Finally, we used S288C *tps1*Δ to evaluate the function of the *MKT1* alleles from each yeast strain—functional alleles of *MKT1* are able to reduce the frequency of persister-like cell formation in S288C *tps1*Δ. Each *MKT1* allele was fully sequenced, and the corresponding amino acid sequence was compared to the reference strain S288C (Supplementary Fig. 6). As observed previously, when the W303 allele of *MKT1* was introduced to the S288C *tps1*Δ strain, formation of persister-like cells was 10-fold less than on YPD (Fig. 3a) and was completely abolished on SD (Fig. 3b) (Gibney *et al.* 2020). Introducing any of the other 4 wild type alleles to S288C also decreased persister-like cell formation in S288C *tps1*Δ. These results suggest that persister-like cell formation in these strains is regulated by other factors beyond *MKT1*.

Taken together, it is clear that there are a number of common *tps1*Δ phenotypes shared among the strains tested. For example, none of the strains accumulated trehalose during stationary phase as expected. Most strains accumulated higher levels of glycogen, were sensitive to acute heat shock, exhibited failed meiosis/sporulation, and were unable to grow on fructose. However, for each phenotype there is at least 1 *tps1*Δ strain that maintains a wild type phenotype; these exceptions to the phenotypic rule could be useful tools to examine the genetic underpinnings of each phenotype. Finally, we also demonstrated that the surprising level of glucose growth by these *tps1*Δ mutants is akin to the described persister-like cells. As persister-like cell regulation in these wild strains appears uncoupled to *MKT1*, our results suggest another regulatory mechanism allows some fraction of *tps1*Δ cells to survive and thrive in glucose. It is clear that there are still uncharacterized mechanisms connecting the function of Tps1 to associated phenotypes, and perhaps the genetics of nonlaboratory strains will be useful in filling this knowledge gap.

### Variable restoration of *tps1*Δ phenotypes by metabolically inactive alleles of *TPS1*

While the molecular mechanisms underlying the diverse phenotypes of *tps1*Δ mutants have remained unclear, in addition to the work described above, multiple publications have demonstrated that intracellular trehalose levels are unrelated to a number of these phenotypes. For example, yeast cells provided with intracellular trehalose fail to restore wild type carbon source utilization, sporulation, or heat resistance (Gibney *et al.* 2015). Though notably intracellular trehalose promotes high levels of desiccation tolerance, suggesting that intracellular trehalose does play roles in stabilization of biomolecules in conditions of low water activity (Tapia *et al.* 2015). In the yeast *Magnaporthe grisea*, catalytically inactive alleles of Tps1 unable to produce T6P complemented phenotypes associated with *tps1*Δ, further suggesting that at least some *tps1*Δ phenotypes are unrelated to trehalose content (Wilson *et al.* 2007). We demonstrate that variations in trehalose levels (Fig. 2a) among different yeast strains also fail to correlate with heat sensitivity (Fig. 2c), sporulation efficiency (Fig. 2d), or carbon source utilization (Fig. 2e). To further investigate the pleiotropic nature of *tps1*Δ phenotypes in *S. cerevisiae*, we took advantage of multiple, published allelic variations and tested each of these alleles for their ability to complement loss-of-function phenotypes associated with *tps1*Δ in our laboratory strain of yeast. These catalytically inactive alleles of Tps1 were originally generated in *M. grisea* to disrupt 4 key residues (R22G, Y99V, W108S, and D153G) required for interaction with glucose-6-phosphate in the catalytic site of the enzyme (Wilson *et al.* 2007). We constructed each of those 4 alleles at the homologous amino acid positions in *S. cerevisiae*, along with a fifth allele combining 2 of the mutations as described in *Materials and Methods* (*tps1*^R24G^, *tps1*^Y102V^, *tps1*^W111S^, *tps1*^D156G^, and *tps1*^Y102V, W111S^). For example, based on amino acid alignment, R22 in *M. grisiae* corresponds to R24 in *S. cerevisiae*. We also included a truncated allele of *TPS1* by inserting a stop codon at amino acid 183, resulting in a C-terminal truncation that removes 63% of the Tps1 protein (*tps1*^W183^*). This truncation was originally described as the *cif1* allele, and reportedly exhibited phenotypic variability in different strains based on glucose and fructose growth compared to a complete *TPS1* gene deletion, in addition to wild type sporulation (González *et al.* 1992; Stucka and Blázquez 1993). Finally, we included the *E. coli* homolog of *TPS1*, *otsA*, which has 53% amino acid similarity to Tps1. This allele has been previously used to restore trehalose-6-phosphate synthase activity to yeast cells (Ruhal *et al.* 2013). Further, expression of *E. coli otsA* in W303 *tps1*Δ and CEN.PK *tps1*Δ partially restored glucose growth (Bonini *et al.* 2000; Deroover *et al.* 2016; Vicente *et al.* 2018). Each of these alleles was cloned into a low-copy plasmid and expressed in S288C *tps1*Δ from the strong, constitutive *TDH3* promoter.

**Fig. 4.**
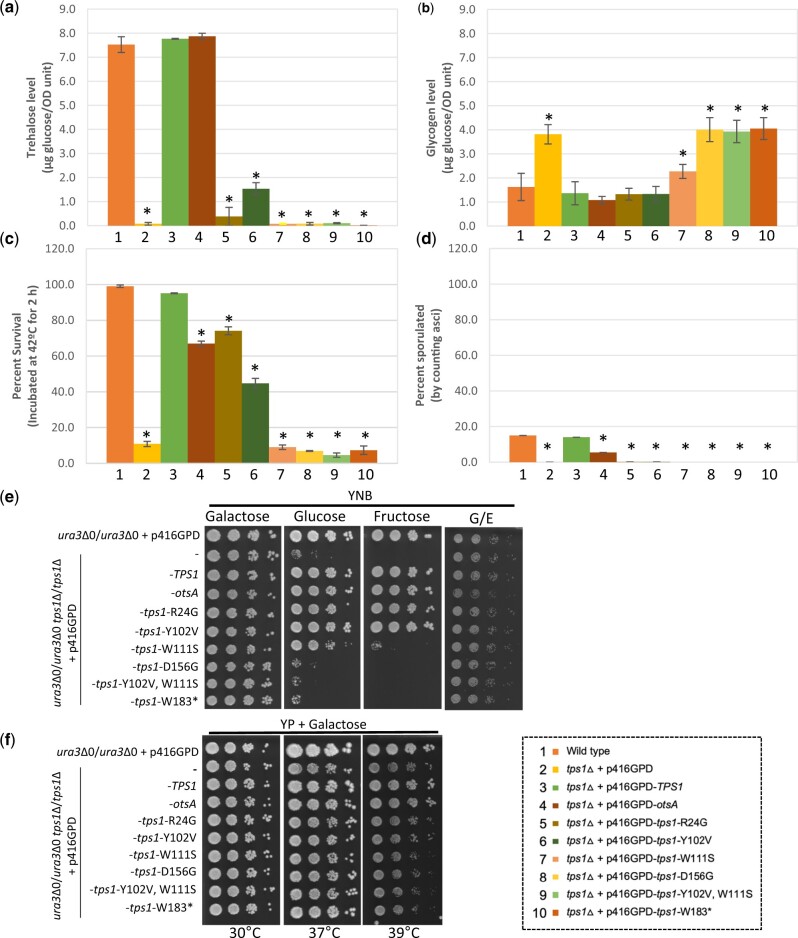
Evaluation of *tps1Δ* phenotypic restoration by complementation with multiple alleles. a) Intracellular trehalose; b) intracellular glycogen; c) thermotolerance; d) sporulation efficiency; e) growth at 30°C for 2 days; f) growth on rich galactose media at 30°C, 37°C and 39°C for 2, 3, and 5 days, respectively. Indicated strains were grown overnight in YNB + 2% galactose liquid before performing assays as described in *Materials and Methods*. For plate images, 10-fold serial dilutions were prepared and spotted onto the indicated media (G/E indicates glycerol/ethanol). The initial dilution had an OD_600_ of 1.0. Three biological replicates were used for all tested phenotypes. Asterisks represent statistical difference (*P* < 0.05) between the mutant alleles and the wild type. Strain legend to the right of panels (e) and (f) applied to (a)–(d).

By measuring trehalose levels, we confirmed that stationary phase *tps1*Δ cells containing the metabolically inactive alleles exhibited severely impaired trehalose production (Fig. 4a). Only Tps1^Y102V^ produced detectable levels of trehalose, though still significantly lower than wild type or complemented *tps1*Δ, producing roughly 20% of wild type trehalose levels. Similar to complementation with plasmid-based wild type *TPS1*, *E. coli otsA* was able to fully restore trehalose to wild type levels as well (Fig. 4a). On the other hand, glycogen levels did not follow the expected trends. As seen in 4 of 5 wild strains, and previously in lab strains, deletion of *TPS1* leads to increased glycogen levels (Fig. 2b) (Parrou *et al.* 1997). Expression of *otsA*, *tps1*^R24G^, and *tps1*^Y102V^ resulted in wild type glycogen levels, despite the 2 metabolically inactive alleles failing to produce trehalose (Fig. 4b). Expression of *tps1*^D156G^, *tps1*^Y102V, W111S^, and *tps1*^W183*^ accumulated similar amounts of glycogen to *tps1*Δ, while *tps1*^W111S^ produced an intermediate level of glycogen that was significantly higher than wild type (Fig. 4b). These results again suggest that metabolic overflow of the shared substrate UDP-glucose does not fully explain the glycogen accumulation phenotypes observed here. Survival after a 2 h heat shock at 42°C was largely uncorrelated with trehalose or glycogen levels, with expression *otsA*, *tps1*^R24G^, and *tps1*^Y102V^ each partially restoring heat resistance, though still significantly less than wild type levels (Fig. 4c). In contrast to glycogen levels and heat survival, none of the *TPS1* alleles were able to fully restore wild type sporulation levels, though *otsA* expression did increase sporulation efficiency to roughly 30% of wild type levels (Fig. 4d). Notably even wild type has lower sporulation efficiency likely due to pre-sporulation culture conditions: to maintain the plasmids, cells were sporulated from minimal medium cultures. Utilization of fermentable carbon sources demonstrated a similar pattern to glycogen accumulation and heat survival, as expression of *otsA*, *tps1*^R24G^, and *tps1*^Y102V^ exhibited wild type growth on glucose and fructose, unlike the other alleles except for *tps1*^W111S^ (Fig. 4e). Expression of *tps1*^W111S^ resulted in an intermediate growth phenotype on glucose and fructose, similar to its intermediate phenotype in glycogen accumulation, despite maintaining a null phenotype for trehalose accumulation and heat survival. Finally, we also examined each allele for the ability to restore wild type growth rates at slightly elevated temperatures (37°C and 39°C). Only expression of *otsA* was able to restore high temperature growth to levels similar to wild type and complemented *tps1*Δ (Fig. 4f). Additionally, our results suggest that the *cif1* allele of *TPS1*, *tps1*-W183*, is nonfunctional and that previous reports of variable growth on glucose may be more related to the *tps1*Δ persister-like phenotype than to any residual metabolic activity of the *cif1* allele (Fig. 4) (Bell *et al.* 1992; Stucka and Blázquez 1993). Taken together, as seen in *M. grisea*, multiple metabolically inactive alleles of *TPS1* are able to partially or fully complement a number of *tps1*Δ phenotypes despite inability to produce trehalose (Table 1). Further, expression of *otsA* results in production of wild type levels of trehalose, restored growth on fructose, but is only partially able to restore high temperature survival and sporulation (Table 1). The observation that *otsA* is able to suppress or partially suppress a number of these phenotypes suggests potential regulatory models: T6P levels, metabolic flux, or UDP-glucose levels may be involved in regulating these phenotypes, though there are likely other possibilities. It is noteworthy that a *tps1*Δ mutant expressing *otsA* still hyperaccumulates sugar-phosphates despite having restored glucose growth, suggesting glycolytic regulation is not completely normal in the *otsA* expression strain (Bonini *et al.* 2000). Considering multiple observations that disconnect trehalose production from a number of trehalose metabolism mutant phenotypes, one possibility is that Tps1, or another protein in trehalose metabolism, has an independent role or roles in the cell. This notion is supported by restoration of carbon source utilization by catalytically inactive variants (R24V and Y102V), and also by the observation that some fraction of Tps1 appears to be free from the rest of the TPS complex (Bell *et al.* 1998). Future studies geared toward using evidence based on natural genetic variants combined with these allelic variations could shed light on the molecular mechanisms underlying these diverse phenotypes.

**Table 1.** Allele-specific complementation of *tps1*Δ phenotypes.

	Wild type	*tps1*Δ + p416GPD-								
		Empty vector	*TPS1*	*otsA*	*tps1*-R24G	*tps1*-Y102V	*tps1*-W111S	*tps1*-D156G	*tps1*-Y102V, W111S	*tps1*-W183*
Glucose growth	+	−/+	+	+	+	+	−/+	−/+	−/+	−/+
Fructose growth	+	−	+	+	+	+	−/+	−	−	−
High temp. growth (39°C)	+	−/+	+	+	−/+	−/+	−/+	−/+	−/+	−/+
High temp. survival (42°C—2h)	+	−	+	−/+	−/+	−/+	−	−	−	−
Sporulation efficiency	+	−	+	−/+	−	−	−	−	−	−
Trehalose level	+	−	+	+	−	−/+	−	−	−	−
Glycogen level	+	++	+	+	+	+	++	++	++	++
*M. grisea* trehalose	+	−	+	Not tested	−	−	−	−	Not tested	Not tested
*M. grisea* sporulation	+	−	+	Not tested	−	+	−	−	Not tested	Not tested
*M. grisea* virulence	+	−	+	−	+	+	−	−	Not tested	Not tested

All scoring is based on Figure 4 data, and each score is relative to wild type levels (+): absent (−), intermediate (−/+), increased (++). *M. grisea* data are from Wilson *et al.*, though the different alleles were genomically integrated rather than being expressed from a plasmid, and the homologous amino acid positions are slightly different in *M. grisea* (R22G, Y99V, W108S, and D153G).

### Construction and phenotypic characterization of *tps2*Δ

*TPS2* encodes the trehalose-6-phosphate phosphatase enzyme, which catalyzes the production of trehalose by dephosphorylation of trehalose-6-phosphate (Fig. 1). Disruption of *TPS2* causes inability to effectively synthesize trehalose and excessive accumulation of the intermediate metabolite trehalose-6-phosphate (de Virgilio *et al.* 1993; Bell *et al.* 1998). Apart from losing metabolic activity, a variety of pleiotropic phenotypes are associated with *tps2*Δ from different genetic backgrounds, including acute thermosensitivity, inability to proliferate at elevated temperature, defective growth on galactose and glycerol/ethanol, and defective in sporulation (de Virgilio *et al.* 1993; Elliott *et al.* 1996; Hohmann *et al.* 1996). As with *tps1*Δ, the mechanism explaining these unrelated phenotypes is not yet elucidated.

**Fig. 5.**
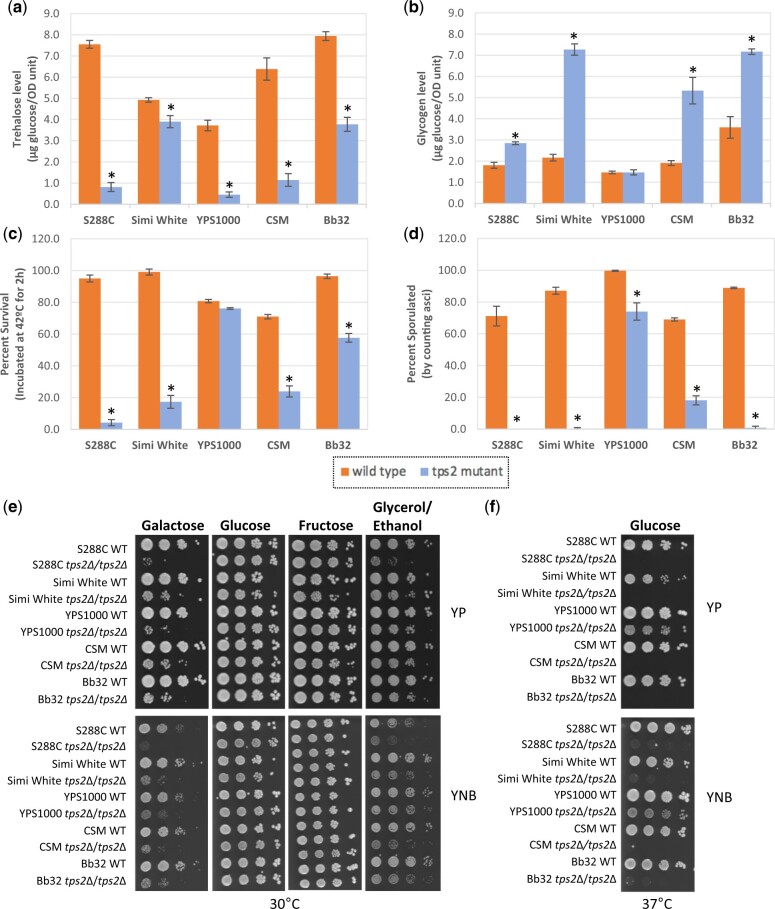
*tps2*Δ mutant phenotypes. a) Intracellular trehalose; b) intracellular glycogen; c) thermotolerance; d) sporulation efficiency; e) growth at 30°C for 2 days; f) growth at 37°C for 3 days. Indicated strains were grown overnight in YNB + 2% glucose liquid before performing assays as described in *Materials and Methods*. For plate images, 10-fold serial dilutions were prepared and spotted onto the indicated media. The initial dilution had an OD_600_ of 1.0. Three biological replicates were used for all tested phenotypes. Asterisks represent statistical difference (*P* < 0.05) between the mutants and their isogenic wild type strains. The strain legend below (c) and (d) applies to (a)–(d).

We constructed *tps2*Δ mutants in the same 5 strains and subjected them to the same phenotypic tests as performed for *tps1*Δ mutants. All 5 *tps2*Δ strains synthesized lower amount of trehalose than their isogenic wild type, though none exhibited total failure to produce trehalose (Fig. 5a). In fact, some of them accumulated comparatively high levels of trehalose such as the Simi White *tps2*Δ and Bb32 *tps2*Δ, with 79% and 48% trehalose levels compared to their isogenic wild type strains, respectively*.* Trehalose accumulation has also been observed in other strain background *tps2*Δ mutants, including W303, and might be explained by the presence of other nonspecific phosphatases independent of the trehalose synthase complex, hydrolyzing T6P into trehalose with lower efficiency than T6P phosphatase (Bell *et al.* 1998; Ratnakumar and Tunnacliffe 2006). The possibility that other proteins in the TPS complex (Tps1, Tps3, and Tsl1) might contribute to this accumulation was refuted when a residual level of T6P phosphatase activity was found in a quadruple *tps1*Δ*tps2*Δ*tps3*Δ*tsl1*Δ strain (Reinders *et al.* 1997). This further indicates that there should be significant T6P phosphatase capacity *in vivo* to sustain observed trehalose accumulation in *tps2*Δ. Four out of 5 strains accumulated higher levels of glycogen in the absence of *TPS2*, ranging from 336% to 158% of the isogenic wild types, while YPS1000 *tps2*Δ did not (Fig. 5b). Similar to *tps1*Δ observations, intracellular trehalose and glycogen levels are not necessarily anticorrelated. Only Simi White *tps2*Δ was larger in cell size compared to its isogenic wild type, which was also the case with Simi White *tps1*Δ (Supplementary Fig. 2b).

Acute thermosensitivity (42°C for 2 h) in the 5 tested stationary *tps2*Δ strains varied: 4 of 5 strains exhibited significantly lower survival rates than their isogenic wild type, though the degree of survival ranged from 5% to 60% (Fig. 5c). YPS1000 *tps2*Δ heat sensitivity did not significantly differ from its isogenic wild type strain, suggesting genetic variation in this strain background can suppress the typical *tps2*Δ thermosensitivity phenotype (Fig. 5c). Temperature-sensitive growth is another stress phenotype associated with *tps2*Δ in multiple previous studies (Piper and Lockheart 1988). All 5 *tps2*Δ strains observable detectable growth defects at elevated temperature (Fig. 5f and Supplementary Fig. 7). Complete cessation of growth was found in *tps2*Δ strains in the S288C, Simi White, CSM, and Bb32 genetic backgrounds. As with acute thermosensitivity, YPS1000 *tps2*Δ cells were less affected by the elevated temperature and had only slightly smaller colonies compared to wild type at similar dilutions (Fig. 5f and Supplementary Fig. 7). Trehalose, as the end-product of the trehalose biosynthesis pathway, has been long regarded as a chemical chaperone to protect cells against various types of stresses, including heat stress (Hottiger *et al.* 1987; Herdeiro *et al.* 2006; Crowe 2007). However, as observed in *tps1*Δ cells, increased intracellular trehalose accumulation failed to repair *tps2*Δ heat sensitivity, raising the doubt that it can stabilize intact, hydrated cells during thermal stress (Gibney *et al.* 2015). Another hypothesis is that excessive accumulation of T6P at elevated temperature is likely to disturb energy metabolism and might be the cause of thermosensitive growth in *tps2*Δ (Thevelein and Hohmann 1995). However, thermosensitivity was not a general feature of sugar-phosphate accumulation, though it might be a T6P-specific phenotype (Gibney *et al.* 2018). Similar to *tps1*Δ mutants, the mechanism underlying thermosensitivity associated with *tps2*Δ remains unclear.

Another shared phenotype between *tps1*Δ and *tps2*Δ mutants is an inability to sporulate in the S288C background (Sur *et al.* 1994; Gibney *et al.* 2015; Liu *et al.* 2020). Here we similarly show that S288C *tps2*Δ, Simi White *tps2*Δ, and Bb32 *tps2*Δ are unable to sporulate (Fig. 5d). However, compared to their own wild type strains, CSM *tps2*Δ and YPS1000 *tps2*Δ reached 26% and 74% sporulation, respectively (Fig. 5d). As shown in Fig. 2d, YPS1000 *tps1*Δ also exhibited a higher sporulation efficiency compared to those from other genetic backgrounds, suggesting an allelic difference in the gene(s) regulating sporulation of trehalose biosynthesis mutants in YPS1000. Interestingly and in contrast to these results, a *tps2*Δ mutant in the W303 genetic background reportedly exhibited identical sporulation efficiency compared to its wild type (Neves *et al.* 1995; Hohmann *et al.* 1996).

Abnormal growth on respiratory carbon sources has been observed for multiple *tps2*Δ lab strains (S288C, CEN.PK) (Walther *et al.* 2013; Gibney *et al.* 2015). While *tps1*Δ mutants did not exhibit growth defects on respiratory carbon sources (Fig. 2e), all 5 *tps2*Δ mutant strains grew slower than wild type cells on respiratory carbon sources including galactose (preferred respiration substrate) and combined glycerol/ethanol (obligate respiration substrate) (Fig. 5e). On preferentially fermented carbon sources glucose and fructose, all 5 mutants grew similarly to their isogenic wild type strains (Fig. 5e). One model for this respiratory growth impairment is that increased accumulation of T6P in the *tps2*Δ mutant causes an increase in cytosolic pH and therefore strongly reduced growth rates on nonfermentable carbon sources (Walther *et al.* 2013). Evidence supporting this hypothesis is that normal growth was observed in *tps1*Δ*tps2*Δ, which does not produce T6P (Bonini *et al.* 2000). However, despite the observation that T6P inhibits hexokinase *in vitro*, suggesting prevention of uncontrolled sugar phosphorylation and fatal depletion of ATP, yeast cells can tolerate 10-fold decreases and 4-fold increases in T6P without any noticeable impact on glycolytic flux or on the ability to consume fermentable carbon sources (Blázquez *et al.* 1993; Thevelein and Hohmann 1995; Hohmann *et al.* 1996; Walther *et al.* 2013). Further, in a strain lacking *TPS1*, expression of the bacterial T6P synthase *otsA* or the leaky *TPS1* allele (*byp1-3*) resulted in no difference on fermentable carbon source growth or fermentation rate, even though significant amounts of T6P were produced (Hohmann *et al.* 1992, 1996; Bonini *et al.* 2000). Overall, the pleiotropic phenotypes observed in *tps1*Δ and *tps2*Δ further emphasize the crucial, but unclear role of the trehalose biosynthesis pathway in regulation of respiratory and fermentative metabolism.

### Construction and phenotypic characterization of *tps3*Δ, *tsl1*Δ, and *tps3*Δ*tsl1*Δ

In *S. cerevisiae*, 2 additional proteins in the trehalose synthase complex, Tps3 and Tsl1, were identified based on a high degree of amino acid sequence similarity with Tps1 and Tps2, despite not having defined trehalose synthesis catalytic activities. The *TPS3* and *TSL1* genes are paralogs resulting from a whole genome duplication in the *S. cerevisiae* lineage (Ferreira *et al.* 1996; Bell *et al.* 1998). Using the yeast 2-hybrid approach, it was demonstrated that Tsl1 and Tps3 can interact with Tps1 and Tps2 *in vivo*, while the latter 2 proteins also interact with each other (Reinders *et al.* 1997; de Silva-Udawatta and Cannon 2001). These 4 proteins share roughly 33% identity over a stretch of 500 amino acids, but it is not known whether physical interactions between these proteins occur through this common sequence (François and Parrou 2001). *TPS3* and *TSL1* mRNA expression levels indicate that these proteins may be regulated differently under specific growth conditions: *TPS3* is expressed at a constant rate in exponentially growing and stationary-phase cells, while *TSL1* expression is greatly enhanced upon entrance into stationary phase (Winderickx *et al.* 1996). Although *tps3*Δ, *tsl1*Δ, or *tps3*Δ*tsl1*Δ mutants have not been as extensively studied as *tps1*Δ and *tps2*Δ, Tps3 and Tsl1 are seemingly important contributors to trehalose metabolism as both paralogs have been maintained in the yeast genome and both encode proteins that interact with the other trehalose synthesis proteins. Here, we constructed both single deletion mutants and the double deletion mutant in all 5 genetic backgrounds and performed the same phenotype tests used for *tps1*Δ and *tps2*Δ. Most single *tps3*Δ or *tsl1*Δ deletions had a minor effect on trehalose levels, if any (Fig. 6a). However, deleting both *TPS3* and *TSL1* reduced trehalose production to levels ranging from 55.3% to 88.4% of wild type production levels (Fig. 6a). This was also reported in the W303 lab strain where the single deletion of *TPS3* or *TSL1* slightly lowered the trehalose content, and the double deletion had <50% trehalose compared to the wild type strain (Reinders *et al.* 1997; Bell *et al.* 1998). Glycogen levels were slightly higher in S288C, Simi White, and YPS1000 *tsl1*Δ and *tps3*Δ*tsl1*Δ strains compared to their isogenic wild types (Fig. 6b). We did not observe any significant cell size variations with any of these mutants (Supplementary Fig. 2). It is not clear why deleting both of these genes only results in mild to moderate changes to intracellular trehalose content. It is possible that these proteins are not very important for trehalose production, or that we have not identified an appropriate condition that strongly requires one or both of these genes.

**Fig. 6.**
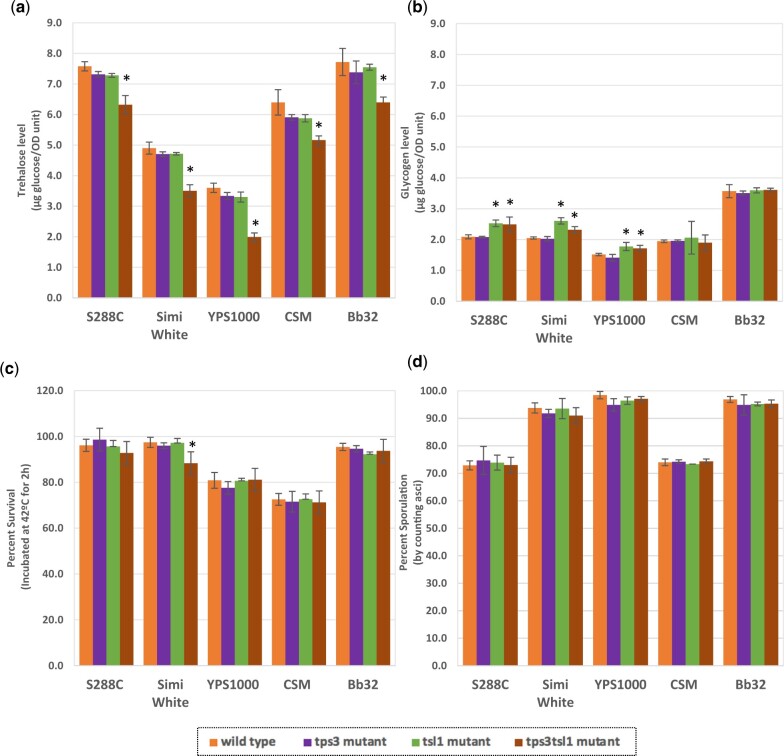
*tps3*Δ, *tsl1*Δ, and *tps3*Δ*tsl*Δ mutant phenotypes. a) Intracellular trehalose; b) intracellular glycogen; c) thermotolerance; d) sporulation efficiency; e) growth at 30°C for 2–3 days; f) growth at 37°C for 3 days. Indicated strains were grown overnight in YNB + 2% glucose liquid before performing assays as described in *Materials and Methods*. For plate images, 10-fold serial dilutions were prepared and spotted onto the indicated media. The initial dilution had an OD_600_ of 1.0. Three biological replicates were used for all tested phenotypes. Asterisks represent statistical difference (*P* < 0.05) between the mutants and their isogenic wild type strains.

**Fig. 6.**
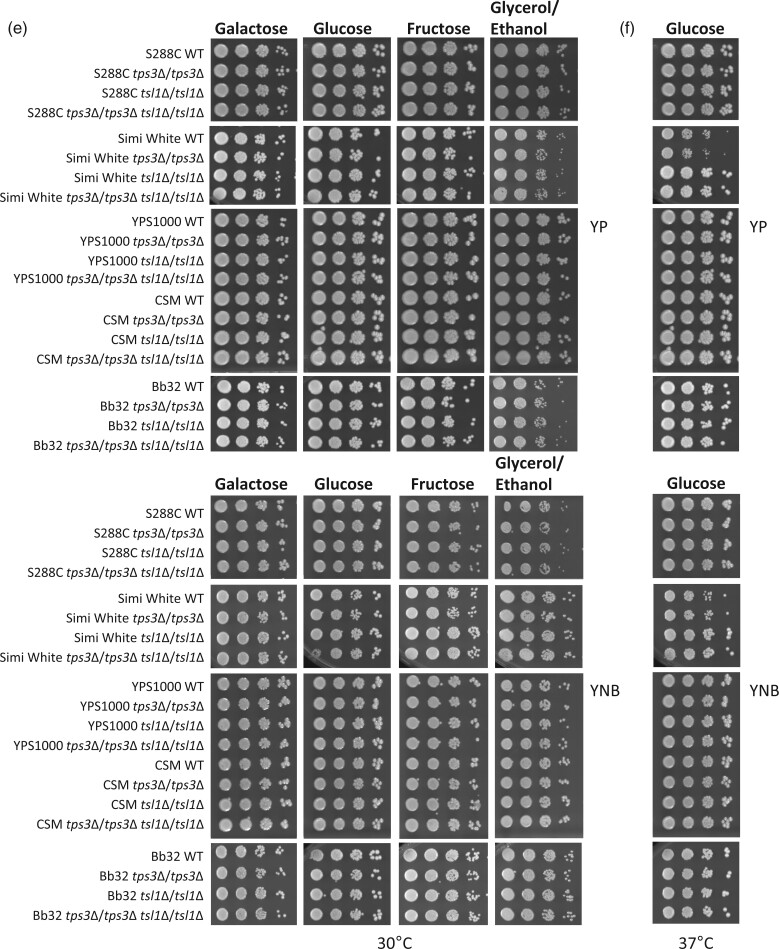
Continued.

Almost every single and double mutant in all 5 strains had wild type levels of thermotolerance when exposed to 42°C for 2 h (Fig. 6c). Only 1 strain, Simi White *tps3*Δ*tsl1*Δ, exhibited mild but statistically significant thermosensitivity (Fig. 6c). It is possible that the slight thermosensitivity of Simi White *tps3*Δ*tsl1*Δ results from destabilized TPS complex, and subsequently developing *tps1*Δ-like thermosensitivity. Notably, in the W303 background after a heat shock, *tps3*Δ*tsl1*Δ cells exhibited moderately reduced Tps1 activity compared to wild type (Reinders *et al.* 1997). This was further supported by another study demonstrating that deletion of *TPS3* and *TSL1* appeared to destabilize the trehalose synthase complex (Bell *et al.* 1998). However, it is also notable that the double deletion and presumably destabilized TPS complex has no effect on thermotolerance in 4 of 5 strains. Similar to observations of thermotolerance, none of the deletion mutants exhibited any growth defect when incubated at high temperature, 37°C (Fig. 6f). Surprisingly, we noticed a mild growth enhancement at 37°C on both rich and minimal media for Simi White *tsl1*Δ and Simi White *tps3*Δ*tsl1*Δ compared to their isogenic wild type and the single *tps3*Δ mutant (Fig. 6f and Supplementary Fig. 7). To further confirm this phenotype, we constructed multiple, independent Simi White *tsl1*Δ deletion mutants by dissecting independent heterozygous transformants, collecting resulting segregants from each dissection, and retesting their ability to grow 37°C. All tested *tsl1*Δ strains exhibited enhanced growth at 37°C compared to wild type (Supplementary Fig. 8). It is possible that a genetic variant between Simi White and these other strains could reveal the genetic basis of this mildly enhanced thermotolerance and illuminate the connections between trehalose metabolism and cellular stress response.

Deleting *TPS*3, *TSL1*, or both did not significantly compromise sporulation efficiency of any tested strain (Fig. 6d). Similarly, none of these mutants in any of these strains exhibited growth defects on tested carbon sources (Fig. 6e). Although there are few published studies examining *tps3*Δ or *tsl1*Δ mutants, a published dissertation demonstrated that in the lab strain CEN.PK, *tps3*Δ, *tsl1*Δ, and *tps3*Δ*tsl1*Δ all exhibited a severe deficiency in ascus formation (Karabulut 2012). This was not the case with the 5 strains tested here, which all had wild type levels of sporulation, suggesting that the CEN.PK phenotypes may be strain-specific and also useful for future studies to investigate the genetic connections between trehalose metabolism and sporulation. Taken together, phenotypes associated with *tps3*Δ, *tsl1*Δ, or *tps3*Δ*tsl1*Δ mutants are mild if present at all. Further work will be required to determine the precise role(s) and significance that these proteins play in trehalose metabolism or other cellular processes.

## Conclusions

In this study, we constructed mutants of the trehalose biosynthesis pathway (*TPS1*, *TPS2*, *TPS3*, and *TSL1*) in 5 diverse *S. cerevisiae* strains to rigorously examine whether published lab strain phenotypes are also exhibited by wild strains. For each mutant, we assessed trehalose production, glycogen production, cell size, acute thermotolerance, high temperature growth, sporulation efficiency, and growth on a variety of carbon sources in rich and minimal medium.

Regarding deletion of *TPS1* and *TPS2*, many reported laboratory phenotypes were observed with some variations in magnitude. However, for most phenotypes at least 1 mutant strain exhibited little to no defect—these variants have potential value to dissect the underlying genetic basis for these phenotypic variations, and further our understanding of how trehalose metabolism is integrated into these diverse cellular processes. We further demonstrated that *otsA* and a subset of published, catalytically inactive alleles of *tps1* are able to selectively complement *tps1*Δ phenotypes (Wilson *et al.* 2007). These observations align with similar findings in *M. grisea*, and further support the notion that trehalose itself does not explain many of the pleiotropic phenotypes associated with *tps1*Δ. We observed very mild, if any, phenotypes associated with deletion of either *TSL1* or *TPS3*. Though the double deletion consistently has moderately impaired trehalose production in each strain, no other strong or even mild phenotype was commonly observed for these mutants, suggesting either a minor role for these proteins, an undefined conditional role, or an undefined role outside of trehalose production. One exception to these observations included a mild resistance to thermal stress in Simi White associated with *tsl1*Δ, though the mechanism behind this phenotype is unknown. Future studies taking advantage of these natural and engineered phenotypic variants could provide deeper mechanistic insight into the multiple cellular roles of the trehalose metabolic pathway.

## Supplementary Material

jkac196_Supplemental_Material

## Data Availability

Strains and plasmids are available upon request. The authors affirm that all data necessary for confirming the conclusions of the article are present within the article, figures, and tables. Supplemental material is available at *G3* online.

## References

[jkac196-B1] Argüelles JC. Heat-shock response in a yeast tps1 mutant deficient in trehalose synthesis. FEBS Lett. 1994;350(2–3):266–270. doi:10.1016/0014-5793(94)00786-1. 8070577

[jkac196-B2] Bell W , Klaassen P, Ohnacker M, Boller T, Herweijer M, Schoppink P, Vanderzee P, Wiemken A. Characterization of the 56‐kDa subunit of yeast trehalose‐6‐phosphate synthase and cloning of its gene reveal its identity with the product of CIF1, a regulator of carbon catabolite inactivation. Eur J Biochem. 1992;209(3):951–959. doi:10.1111/j.1432-1033.1992.tb17368.x. 1425702

[jkac196-B3] Bell W , Sun W, Hohmann S, Wera S, Reinders A, de Virgilio C, Wiemken A, Thevelein JM. Composition and functional analysis of the *Saccharomyces cerevisiae* trehalose synthase complex. J Biol Chem. 1998;273(50):33311–33319. doi:10.1074/jbc.273.50.33311. 9837904

[jkac196-B4] Blázquez MA , Lagunas R, Gancedo C, Gancedo JM. Trehalose-6-phosphate, a new regulator of yeast glycolysis that inhibits hexokinases. FEBS Lett. 1993;329(1–2):51–54. doi:10.1016/0014–5793(93)80191-V. 8354408 10.1016/0014-5793(93)80191-v

[jkac196-B5] Bonini BM , Van Dijck P, Thevelein JM. Uncoupling of the glucose growth defect and the deregulation of glycolysis in *Saccharomyces cerevisiae* tps1 mutants expressing trehalose-6-phosphate-insensitive hexokinase from *Schizosaccharomyces pombe*. Biochim Biophys Acta. 2003;1606:83–93. doi:10.1016/S0005-2728(03)00086-0. 14507429 10.1016/s0005-2728(03)00086-0

[jkac196-B6] Bonini BM , Van Vaeck C, Larsson C, Gustafsson L, Ma P, Winderickx J, Van Dijck P, Thevelein JM. Expression of *Escherichia coli* otsA in a *Saccharomyces cerevisiae* tps1 mutant restores trehalose 6-phosphate levels and partly restores growth and fermentation with glucose and control of glucose influx into glycolysis. Biochem J. 2000;350(1):261–268. doi:10.1042/0264-6021:3500261. 10926852 PMC1221250

[jkac196-B7] Borneman AR , Forgan AH, Kolouchova R, Fraser JA, Schmidt SA. Whole genome comparison reveals high levels of inbreeding and strain redundancy across the spectrum of commercial wine strains of *Saccharomyces cerevisiae*. G3 (Bethesda). 2016;6:957–974. doi:10.1534/g3.115.025692. 26869621 PMC4825664

[jkac196-B8] Botstein D , Fink GR. Yeast: an experimental organism for 21st century biology. Genetics. 2011;189(3):695–704. doi:10.1534/genetics.111.130765. 22084421 PMC3213361

[jkac196-B9] Brachmann CB , Davies A, Cost GJ, Caputo E, Li J, Hieter P, Boeke JD. Designer deletion strains derived from *Saccharomyces cerevisiae* S288C: a useful set of strains and plasmids for PCR-mediated gene disruption and other applications. Yeast. 1998;14(2):115–132. doi:10.1002/(SICI)1097-0061(19980130)14:2<115::AID-YEA204>3.0.CO;2-2. 9483801 10.1002/(SICI)1097-0061(19980130)14:2<115::AID-YEA204>3.0.CO;2-2

[jkac196-B10] Byrne KP , Wolfe KH. The Yeast Gene Order Browser: combining curated homology and syntenic context reveals gene fate in polyploid species. Genome Res. 2005;15(10):1456–1461. doi:10.1101/gr.3672305. 16169922 PMC1240090

[jkac196-B11] Calahan D , Dunham M, Desevo C, Koshland DE. Genetic analysis of desiccation tolerance in *Sachharomyces cerevisiae*. Genetics. 2011;189(2):507–519. doi:10.1534/genetics.111.130369. 21840858 PMC3189811

[jkac196-B12] Cannon JF , Pringle JR, Fiechter A, Khalil M. Characterization of glycogen-deficient glc mutants of *Saccharomyces cerevisiae*. Genetics. 1994;136(2):485–503. doi:10.1093/genetics/136.2.485. 8150278 PMC1205803

[jkac196-B13] Chin BL , Ryan O, Lewitter F, Boone C, Fink GR. Genetic variation in *Saccharomyces cerevisiae*: circuit diversification in a signal transduction network. Genetics. 2012;192(4):1523–1532. doi:10.1534/genetics.112.145573. 23051644 10.1534/genetics.112.145573 PMC3512157

[jkac196-B14] Crowe J. H. Trehalose as a “chemical chaperone”: fact and fantasy. Adv Exp Med Biol. 2007;594:143–158. doi:10.1007/978-0-387–39975-1_13. 17205682 10.1007/978-0-387-39975-1_13

[jkac196-B15] de Mesquita JF , Panek AD, de Araujo PS. In silico and in vivo analysis reveal a novel gene in *Saccharomyces cerevisiae* trehalose metabolism. BMC Genomics. 2003;4(1):45. doi:10.1186/1471–2164-4–45. 14614785 10.1186/1471-2164-4-45 PMC280675

[jkac196-B16] de Silva-Udawatta MN , Cannon JF. Roles of trehalose phosphate synthase in yeast glycogen metabolism and sporulation. Mol Microbiol. 2001;40(6):1345–1356. doi:10.1046/j.1365-2958.2001.02477.x. 11442833

[jkac196-B17] de Virgilio C , Burckert N, Bell W, Jeno P, Boller T, Wiemken A. Disruption of TPS2, the gene encoding the 100‐kDa subunit of the trehalose‐6‐phosphate synthase/phosphatase complex in *Saccharomyces cerevisiae*, causes accumulation of trehalose‐6‐phosphate and loss of trehalose‐6‐phosphate phosphatase activity. Eur J Biochem. 1993;212(2):315–323. doi:10.1111/j.1432-1033.1993.tb17664.x. 8444170 10.1111/j.1432-1033.1993.tb17664.x

[jkac196-B18] Deroover S , Ghillebert R, Broeckx T, Winderickx J, Rolland F. Trehalose-6-phosphate synthesis controls yeast gluconeogenesis downstream and independent of SNF1. FEMS Yeast Res. 2016;16(4):fow036. doi:10.1093/femsyr/fow036. 27189362

[jkac196-B19] Duina AA , Miller ME, Keeney JB. Budding yeast for budding geneticists: a primer on the *Saccharomyces cerevisiae* model system. Genetics. 2014;197(1):33–48. doi:10.1534/genetics.114.163188. 24807111 10.1534/genetics.114.163188 PMC4012490

[jkac196-B20] Dunn B , Levine R. P, Sherlock G. Microarray karyotyping of commercial wine yeast strains reveals shared, as well as unique, genomic signatures. BMC Genomics. 2005;6:53. doi:10.1186/1471–2164-6–53. 15833139 10.1186/1471-2164-6-53 PMC1097725

[jkac196-B21] Elbein AD , Pan YT, Pastuszak I, Carroll D. New insights on trehalose: a multifunctional molecule. Glycobiology. 2003;13(4):17R–127. doi:10.1093/glycob/cwg047. 12626396

[jkac196-B22] Eleutherio ECA , Araujo PS, Panek AD. Protective role of trehalose during heat stress in *Saccharomyces cerevisiae*. Cryobiology. 1993;30(6):591–596. doi:10.1006/cryo.1993.1061. 8306706

[jkac196-B23] Elliott B , Haltiwanger RS, Futcher B. Synergy between trehalose and Hsp104 for thermotolerance in *Saccharomyces cerevisiae*. Genetics. 1996;144(3):923–933. doi:10.1093/genetics/144.3.923. 8913738 PMC1207632

[jkac196-B24] Engel SR , Dietrich FS, Fisk DG, Binkley G, Balakrishnan R, Costanzo MC, Dwight SS, Hitz BC, Karra K, Nash RS, et al The reference genome sequence of *Saccharomyces cerevisiae*: then and now. G3 (Bethesda). 2014;4:389–398. doi:10.1534/g3.113.008995. 24374639 PMC3962479

[jkac196-B25] Enyenihi AH , Saunders WS. Large-scale functional genomic analysis of sporulation and meiosis in *Saccharomyces cerevisiae*. Genetics. 2003;163(1):47–54. doi:10.1093/genetics/163.1.47. 12586695 PMC1462418

[jkac196-B26] Ferreira JC , Suva JT, Panek AD. A regulatory role for TSL1 on trehalose synthase activity. Biochem Mol Biol Int. 1996;38(2):259–265. 8850521

[jkac196-B27] François J , Parrou JL. Reserve carbohydrates metabolism in the yeast *Saccharomyces cerevisiae*. FEMS Microbiol Rev. 2001;25(1):125–145. doi:10.1111/j.1574-6976.2001.tb00574.x. 11152943

[jkac196-B28] Gallone B , Steensels J, Prahl T, Soriaga L, Saels V, Herrera-Malaver B, Merlevede A, Roncoroni M, Voordeckers K, Miraglia L, et al Domestication and divergence of *Saccharomyces cerevisiae* beer yeasts. Cell. 2016;166(6):1397–1410.e16. doi:10.1016/j.cell.2016.08.020. 27610566 10.1016/j.cell.2016.08.020 PMC5018251

[jkac196-B29] Gancedo C , Flores CL. The importance of a functional trehalose biosynthetic pathway for the life of yeasts and fungi. FEMS Yeast Res. 2004;4(4–5):351–359. doi:10.1016/S1567-1356(03)00222-8. 14734015

[jkac196-B30] Giaever G , Nislow C. The yeast deletion collection: a decade of functional genomics. Genetics. 2014;197(2):451–465. doi:10.1534/genetics.114.161620. 24939991 PMC4063906

[jkac196-B31] Gibney PA , Chen A, Schieler A, Chen JC, Xu Y, Hendrickson DG, Scott McIsaac R, Rabinowitz JD, Botstein D. A tps1Δ persister-like state in *Saccharomyces cerevisiae* is regulated by MKT1. PLoS One. 2020;15(5):e0233779. doi:10.1371/journal.pone.0233779. 32470059 PMC7259636

[jkac196-B32] Gibney PA , Schieler A, Chen JC, Bacha-Hummel JM, Botstein M, Volpe M, Silverman SJ, Xu Y, Bennett BD, Rabinowitz JD, et al Common and divergent features of galactose-1-phosphate and fructose-1-phosphate toxicity in yeast. MBoC. 2018;29(8):897–910. doi:10.1091/mbc.E17-11-0666. 29444955 PMC5896929

[jkac196-B33] Gibney PA , Schieler A, Chen JC, Rabinowitz JD, Botstein D. Characterizing the in vivo role of trehalose in *Saccharomyces cerevisiae* using the AGT1 transporter. Proc Natl Acad Sci USA. 2015;112(19):6116–6121. doi:10.1073/pnas.1506289112. 25918382 PMC4434743

[jkac196-B34] Gibson DG , Young L, Chuang RY, Venter JC, Hutchison CA, Smith HO. Enzymatic assembly of DNA molecules up to several hundred kilobases. Nat Methods. 2009;6(5):343–345. doi:10.1038/nmeth.1318. 19363495

[jkac196-B35] Goffeau A , Barrell G, Bussey H, Davis RW, Dujon B, Feldmann H, Galibert F, Hoheisel JD, Jacq C, Johnston M, et al Life with 6000 genes. Science. 1996;274(5287):546–567. doi:10.1126/science.274.5287.546. 8849441 10.1126/science.274.5287.546

[jkac196-B36] González MI , Blázquez MA, Gancedo C, Stucka R, Feldmann H. Molecular cloning of CIF1, a yeast gene necessary for growth on glucose. Yeast. 1992;8(3):183–192. doi:10.1002/yea.320080304. 1315471

[jkac196-B37] Guillou V , Plourde-Owobi L, Parrou JL, Goma G, François J. Role of reserve carbohydrates in the growth dynamics of *Saccharomyces cerevisiae*. FEMS Yeast Res. 2004;4(8):773–787. doi:10.1016/j.femsyr.2004.05.005. 15450184 10.1016/j.femsyr.2004.05.005

[jkac196-B38] Herdeiro RS , Pereira MD, Panek AD, Eleutherio ECA. Trehalose protects *Saccharomyces cerevisiae* from lipid peroxidation during oxidative stress. Biochim Biophys Acta. 2006;1760(3):340–346. doi:10.1016/j.bbagen.2006.01.010. 16510250

[jkac196-B39] Hittinger CT. Saccharomyces diversity and evolution: a budding model genus. Trends Genet. 2013;29(5):309–317. doi:10.1016/j.tig.2013.01.002. 23395329 10.1016/j.tig.2013.01.002

[jkac196-B40] Hohmann S , Bell W, Neves MJ, Valckx D, Thevelein JM. Evidence for trehalose-6-phosphate-dependent and-independent mechanisms in the control of sugar influx into yeast glycolysis. Mol Microbiol. 1996;20(5):981–991. doi:10.1111/j.1365–2958.1996.tb02539.x. 8809751 10.1111/j.1365-2958.1996.tb02539.x

[jkac196-B41] Hohmann S , Huse K, Valentin E, Mbonyi K, Thevelein JM, Zimmermann FK. Glucose-induced regulatory defects in the *Saccharomyces cerevisiae* byp1 growth initiation mutant and identification of MIG1 as a partial suppressor. J Bacteriol. 1992;174(12):4183–4188. doi:10.1128/jb.174.12.4183-4188.1992. 1597433 PMC206133

[jkac196-B42] Hohmann S , Neves MJ, de Koning W, Alijo R, Ramos J, Thevelein JM. The growth and signalling defects of the ggs1 (fdp1/byp1) deletion mutant on glucose are suppressed by a deletion of the gene encoding hexokinase PII. Curr Genet. 1993;23(4):281–289. doi:10.1007/BF00310888. 8467527

[jkac196-B43] Hohmann S , Van Dijck P, Luyten K, Thevelein JM. The byp1-3 allele of the *Saccharomyces cerevisiae* GGS1/TPS1 gene and its multi-copy suppressor tRNAGLN (CAG): ggs1/Tps1 protein levels restraining growth on fermentable sugars and trehalose accumulation. Curr Genet. 1994;26(4):295–301. doi:10.1007/BF00310492. 7882422

[jkac196-B44] Hottiger T , Boller T, Wiemken A. Correlation of trenalose content and heat resistance in yeast mutants altered in the RAS/adenylate cyclase pathway: is trehalose a thermoprotectant? FEBS Lett. 1989;255(2):431–434. doi:10.1016/0014–5793(89)81139-1. 2676607 10.1016/0014-5793(89)81139-1

[jkac196-B45] Hottiger T , Schmutz P, Wiemken A. Heat-induced accumulation and futile cycling of trehalose in *Saccharomyces cerevisiae*. J Bacteriol. 1987;169(12):5518–5522. doi:10.1128/jb.169.12.5518-5522.1987. 2960663 PMC213980

[jkac196-B46] Jain NK , Roy I. Effect of trehalose on protein structure. Protein Sci. 2009;18:24–36. doi:10.1002/pro.3. 19177348 PMC2708026

[jkac196-B47] Karabulut Ş. First steps towards a better understanding of the function of the TPS complex from *Saccharomyces cerevisiae*, 2012. http://hdl.handle.net/11527/2839.

[jkac196-B48] Kellis M , Patterson N, Endrizzi M, Birren B, Lander ES. Sequencing and comparison of yeast species to identify genes and regulatory elements. Nature. 2003;423(6937):241–254. doi:10.1038/nature01644. 12748633

[jkac196-B49] Liti G , Carter DM, Moses AM, Warringer J, Parts L, James SA, Davey RP, Roberts IN, Burt A, Koufopanou V, et al Population genomics of domestic and wild yeasts. Nature. 2009;458(7236):337–341. doi:10.1038/nature07743. 19212322 PMC2659681

[jkac196-B50] Liu Y , Wood NE, Marchand AJ, Arguello-Miranda O, Doncic A. Functional interrelationships between carbohydrate and lipid storage, and mitochondrial activity during sporulation in *Saccharomyces cerevisiae*. Yeast. 2020;37(3):269–279. doi:10.1002/yea.3460. 31960994

[jkac196-B51] Londesborough J , Vuorio OE. Purification of trehalose synthase from baker’s yeast: its temperature‐dependent activation by fructose 6‐phosphate and inhibition by phosphate. Eur J Biochem. 1993;216(3):841–848. doi:10.1111/j.1432-1033.1993.tb18206.x. 8404904 10.1111/j.1432-1033.1993.tb18206.x

[jkac196-B52] Luyten K , Albertyn J, Skibbe WF, Prior BA, Ramos J, Thevelein JM, Hohmann S. Fps1, a yeast member of the MIP family of channel proteins, is a facilitator for glycerol uptake and efflux and is inactive under osmotic stress. EMBO J. 1995;14:1360–1371. doi:10.1002/j.1460-2075.1995.tb07122.x. 7729414 PMC398221

[jkac196-B53] Mahmud SA , Hirasawa T, Shimizu H. Differential importance of trehalose accumulation in *Saccharomyces cerevisiae* in response to various environmental stresses. Journal of Bioscience and Bioengineering. 2010;109(3):262–266. doi:10.1016/j.jbiosc.2009.08.500. 20159575 10.1016/j.jbiosc.2009.08.500

[jkac196-B54] Mortimer RK , Johnston JR Genealogy of principal strains of the yeast genetic stock center. Genetics. 1986;113(1):35–43. doi:10.1093/genetics/113.1.35. 3519363 PMC1202798

[jkac196-B55] Mumberg D , Müller R, Funk M. Yeast vectors for the controlled expression of heterologous proteins in different genetic backgrounds. Gene. 1995;156(1):119–122. doi:10.1016/0378-1119(95)00037-7. 7737504 10.1016/0378-1119(95)00037-7

[jkac196-B56] Navon G , Shulman RG, Yamane T, Eccleshall TR, Lam KB, Baronofsky JJ, Marmur J, Lam KB. Phosphorus-31 nuclear magnetic resonance studies of wild-type and glycolytic pathway mutants of *Saccharomyces cerevisiae*. Biochemistry. 1979;18(21):4487–4499. doi:10.1021/bi00588a006. 40590

[jkac196-B57] Neves MJ , Hohmann S, Bell W, Dumortier F, Luyten K, Ramos J, Cobbaert P, de Koning W, Kaneva Z, Thevelein JM. Control of glucose influx into glycolysis and pleiotropic effects studied in different isogenic sets of *Saccharomyces cerevisiae* mutants in trehalose biosynthesis. Curr Genet. 1995;27(2):110–122. doi:10.1007/BF00313424. 7788713 10.1007/BF00313424

[jkac196-B58] Panek AC , de Araujo PS, Moura Neto V, Panek AD. Regulation of the trehalose-6-phosphate synthase complex in Saccharomyces - I. Interconversion of forms by phosphorylation. Curr Genet. 1987;11(6–7):459–465. doi:10.1007/BF00384607. 2967122

[jkac196-B59] Parrou JL , François J. A simplified procedure for a rapid and reliable assay of both glycogen and trehalose in whole yeast cells. Anal Biochem. 1997;248(1):186–188. doi:10.1006/abio.1997.2138. 9177741

[jkac196-B60] Parrou JL , Teste MA, François J. Effects of various types of stress on the metabolism of reserve carbohydrates in *Saccharomyces cerevisiae*: Genetic evidence for a stress-induced recycling of glycogen and trehalose. Microbiology. 1997;143(6):1891–1900. doi:10.1099/00221287-143-6-1891. 9202465

[jkac196-B61] Peeters K , van Leemputte F, Fischer B, Bonini BM, Quezada H, Tsytlonok M, Haesen D, Vanthienen W, Bernardes N, Gonzalez-Blas CB, et al Fructose-1,6-bisphosphate couples glycolytic flux to activation of Ras. Nat Commun. 2017;8(1). Article number: 922. doi:10.1038/s41467-017-01019-z. PMC5640605 29030545

[jkac196-B62] Peter J , De Chiara M, Friedrich A, Yue JX, Pflieger D, Bergström A, Sigwalt A, Barre B, Freel K, Llored A, et al Genome evolution across 1,011 *Saccharomyces cerevisiae* isolates. Nature. 2018;556(7701):339–344. doi:10.1038/s41586-018-0030-5. 29643504 PMC6784862

[jkac196-B63] Petri JR Eine kleine Modification des Koch’schen Plattenverfahrens (A minor modification of the pating technique of Koch). Central. Bacteriol Parasit. 1887;1:279–280.

[jkac196-B64] Piper PW , Lockheart A A temperature-sensitive mutant of *Sacchromyces cerevisiae* defective in the specific phosphatase of trehalose biosynthesis. FEMS Microbiol Let. 1988;49(2):245–250. doi:10.1111/j.1574-6968.1988.tb02724.x.

[jkac196-B65] Ratnakumar S , Tunnacliffe A Intracellular trehalose is neither necessary nor sufficient for desiccation tolerance in yeast. FEMS Yeast Res. 2006;6(6):902–913. doi:10.1111/j.1567-1364.2006.00066.x. 16911512 10.1111/j.1567-1364.2006.00066.x

[jkac196-B66] Reinders A , Bürckert N, Hohmann S, Thevelein JM, Boller T, Wiemken A, De Virgilio C Structural analysis of the subunits of the trehalose-6-phosphate synthase/phosphatase complex in *Saccharomyces cerevisiae* and their function during heat shock. Mol Microbiol. 1997;24(4):687–696. doi:10.1046/j.1365–2958.1997.3861749.x. 9194697 10.1046/j.1365-2958.1997.3861749.x

[jkac196-B67] Ruderfer DM , Pratt SC, Seidel HS, Kruglyak L Population genomic analysis of outcrossing and recombination in yeast. Nat Genet. 2006;38(9):1077–1081. doi:10.1038/ng1859. 16892060

[jkac196-B68] Ruhal R , Kataria R, Choudhury B Trends in bacterial trehalose metabolism and significant nodes of metabolic pathway in the direction of trehalose accumulation. Microbial Biotechnol. 2013;6(5):493–502. doi:10.1111/1751-7915.12029. PMC3918152 23302511

[jkac196-B69] Shi L , Sutter BM, Ye X, Tu BP Trehalose is a key determinant of the quiescent metabolic state that fuels cell cycle progression upon return to growth. Mol Biol Cell. 2010;21(12):1982–1990. doi:10.1091/mbc.E10-01-0056. 20427572 PMC2883942

[jkac196-B70] Sikorski RS , Hieter P A system of shuttle vectors and yeast host strains designed for efficient manipulation of DNA in *Saccharomyces cerevisiae*. Genetics. 1989;122(1):19–27. doi:10.1093/genetics/122.1.19. 2659436 PMC1203683

[jkac196-B71] Singer MA , Lindquist S Multiple effects of trehalose on protein folding in vitro and in vivo. Mol Cell. 1998a;1(5):639–648. doi:10.1016/S1097-2765(00)80064-7. 9660948

[jkac196-B72] Singer MA , Lindquist S Thermotolerance in *Saccharomyces cerevisiae*: the Yin and Yang of trehalose. Trends Biotechnol. 1998b;16(11):460–468. doi:10.1016/S0167-7799(98)01251-7. 9830154

[jkac196-B73] Stucka R , Blázquez MA The fdp1 and cif1 mutations are caused by different single nucleotide changes in the yeast CIF1 gene. FEMS Microbiol. Lett. 1993;107(2–3):251–253. doi:10.1111/j.1574–6968.1993.tb06038.x. 8472906 10.1111/j.1574-6968.1993.tb06038.x

[jkac196-B74] Sur IP , Lobo Z, Maitra PK Analysis of PFK3—a gene involved in particulate phosphofructokinase synthesis reveals additional functions of TPS2 in *Saccharomyces cerevisiae*. Yeast. 1994;10(2):199–209. doi:10.1002/yea.320100207. 8203161

[jkac196-B75] Tapia H , Young L, Fox D, Bertozzi CR, Koshland D Increasing intracellular trehalose is sufficient to confer desiccation tolerance to *Saccharomyces cerevisiae*. Proc Natl Acad Sci USA. 2015;112(19):6122–6127. doi:10.1073/pnas.1506415112. 25918381 PMC4434740

[jkac196-B76] Thevelein JM , Hohmann S Trehalose synthase: guard to the gate of glycolysis in yeast? Trends Biochem Sci. 1995;20(1):3–10. doi:10.1016/S0968-0004(00)88938-0. 7878741

[jkac196-B77] Trevisol ETV , Panek AD, de Mesquita JF, Eleutherio ECA Regulation of the yeast trehalose-synthase complex by cyclic AMP-dependent phosphorylation. Biochim Biophys Acta. 2014;1840(6):1646–1650. doi:10.1016/j.bbagen.2013.12.010. 24380875

[jkac196-B78] Van Aelst L , Hohmann S, Bulaya B, de Koning W, Sierkstra L, Neves MJ, Luyten K, Alijo R, Ramos J, Coccetti P, et al Molecular cloning of a gene involved in glucose sensing in the yeast *Saccharomyces cerevisiae*. Mol Microbiol. 1993;8(5):927–943. doi:10.1111/j.1365-2958.1993.tb01638.x. 8355617 10.1111/j.1365-2958.1993.tb01638.x

[jkac196-B79] Van Heerden JH , Wortel MT, Bruggeman FJ, Heijnen JJ, Bollen YJM, Planqué R, Hulshof J, O’Toole TG, Wahl SA, Teusink B Lost in transition: start-up of glycolysis yields subpopulations of nongrowing cells. Science. 2014;343(6174): doi:10.1126/science.1245114. 10.1126/science.1245114 24436182

[jkac196-B80] van Leemputte F , Vanthienen W, Wijnants S, van Zeebroeck G, Thevelein JM Aberrant intracellular pH regulation limiting glyceraldehyde3-phosphate dehydrogenase activity in the glucose-sensitive yeast tps1Δ mutant. MBio. 2020;11(5):e02199–021200. doi:10.1128/mBio.02199-20. 33109759 PMC7593968

[jkac196-B81] Vandercammen A , François J, Hers H Characterization of trehalose‐6‐phosphate synthase and trehalose‐6‐phosphate phosphatase of *Saccharomyces cerevisiae*. Eur J Biochem. 1989;182(3):613–620. doi:10.1111/j.1432-1033.1989.tb14870.x. 2546763

[jkac196-B82] Vicente RL , Spina L, Gómez JPL, Dejean S, Parrou JL, François JM Trehalose-6-phosphate promotes fermentation and glucose repression in *Saccharomyces cerevisiae*. Microb Cell. 2018;5(10):444–459. doi:10.15698/mic2018.10.651. 30386789 PMC6206404

[jkac196-B83] Voit EO Biochemical and genomic regulation of the trehalose cycle in yeast: review of observations and canonical model analysis. Journal of Theoretical Biology. 2003;223(1):55–78. doi:10.1016/S0022-5193(03)00072-9. 12782117 10.1016/s0022-5193(03)00072-9

[jkac196-B84] Vuorio OE , Kalkkinen N, Londesborough J Cloning of two related genes encoding the 56‐kDa and 123‐kDa subunits of trehalose synthase from the yeast *Saccharomyces cerevisiae*. Eur J Biochem. 1993;216(3):849–861. doi:10.1111/j.1432-1033.1993.tb18207.x. 8404905 10.1111/j.1432-1033.1993.tb18207.x

[jkac196-B85] Walther T , Mtimet N, Alkim C, Vax A, Loret MO, Ullah A, Gancedo C, Smits GJ, François JM Metabolic phenotypes of *Saccharomyces cerevisiae* mutants with altered trehalose 6-phosphate dynamics. Biochem. J. 2013;454(2):227–237. doi:10.1042/BJ20130587. 23763276

[jkac196-B86] Wilson RA , Jenkinson JM, Gibson RP, Littlechild JA, Wang ZY, Talbot NJ Tps1 regulates the pentose phosphate pathway, nitrogen metabolism and fungal virulence. EMBO J. 2007;26(15):3673–3685. doi:10.1038/sj.emboj.7601795. 17641690 PMC1949003

[jkac196-B87] Winderickx J , De Winde JH, Crauwels M, Hino A, Hohmann S, Van Dijck P, Thevelein JM Regulation of genes encoding subunits of the trehalose synthase complex in *Saccharomyces cerevisiae*: novel variations of STRE-mediated transcription control? Mol Gen Genet. 1996;252:470–482. doi:10.1007/s004380050252. 8879249

[jkac196-B88] Yi C , Wang F, Dong S, Li H Changes of trehalose content and expression of relative genes during the bioethanol fermentation by *Saccharomyces cerevisiae*. Can J Microbiol. 2016;62(10):827–835. doi:10.1139/cjm-2015-0832. 27510429

