# Supplementary material for: Characterizing phenotypic diversity of trehalose biosynthesis mutants in multiple wild strains of Saccharomyces cerevisiae
Source: G3 (Bethesda). 2022 Aug 5;12(11):jkac196. doi: 10.1093/g3journal/jkac196 (PMC9635654; doi:10.1093/g3journal/jkac196)
Supplement: jkac196_Supplemental_Material [file jkac196_supplemental_material.pptx]

## Slide 1
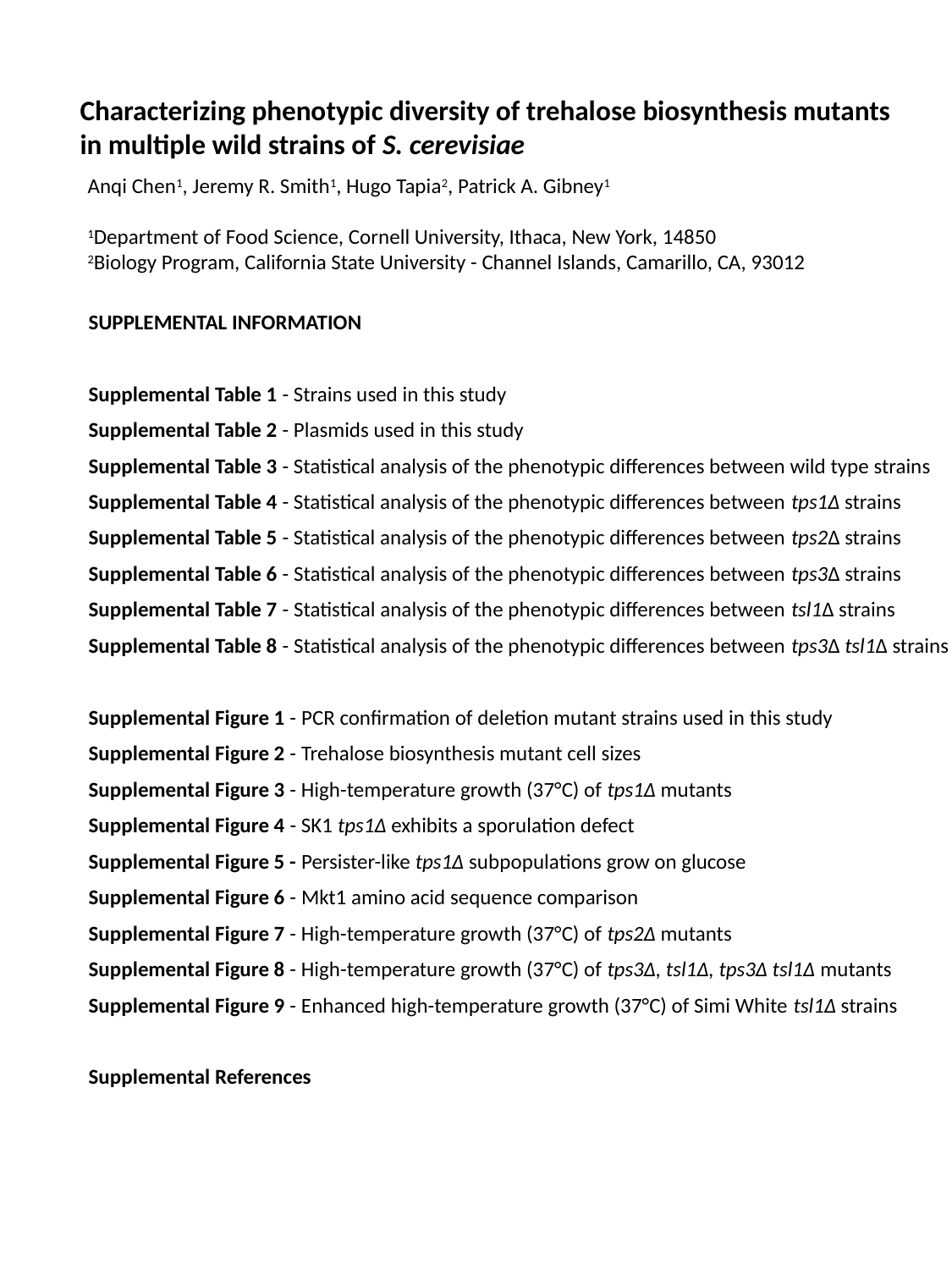

Characterizing phenotypic diversity of trehalose biosynthesis mutants in multiple wild strains of S. cerevisiae
Anqi Chen1, Jeremy R. Smith1, Hugo Tapia2, Patrick A. Gibney1
1Department of Food Science, Cornell University, Ithaca, New York, 14850
2Biology Program, California State University - Channel Islands, Camarillo, CA, 93012
SUPPLEMENTAL INFORMATION
Supplemental Table 1 - Strains used in this study
Supplemental Table 2 - Plasmids used in this study
Supplemental Table 3 - Statistical analysis of the phenotypic differences between wild type strains
Supplemental Table 4 - Statistical analysis of the phenotypic differences between tps1Δ strains
Supplemental Table 5 - Statistical analysis of the phenotypic differences between tps2Δ strains
Supplemental Table 6 - Statistical analysis of the phenotypic differences between tps3Δ strains
Supplemental Table 7 - Statistical analysis of the phenotypic differences between tsl1Δ strains
Supplemental Table 8 - Statistical analysis of the phenotypic differences between tps3Δ tsl1Δ strains
Supplemental Figure 1 - PCR confirmation of deletion mutant strains used in this study
Supplemental Figure 2 - Trehalose biosynthesis mutant cell sizes
Supplemental Figure 3 - High-temperature growth (37°C) of tps1Δ mutants
Supplemental Figure 4 - SK1 tps1Δ exhibits a sporulation defect
Supplemental Figure 5 - Persister-like tps1Δ subpopulations grow on glucose
Supplemental Figure 6 - Mkt1 amino acid sequence comparison
Supplemental Figure 7 - High-temperature growth (37°C) of tps2Δ mutants
Supplemental Figure 8 - High-temperature growth (37°C) of tps3Δ, tsl1Δ, tps3Δ tsl1Δ mutants
Supplemental Figure 9 - Enhanced high-temperature growth (37°C) of Simi White tsl1Δ strains
Supplemental References

## Slide 2
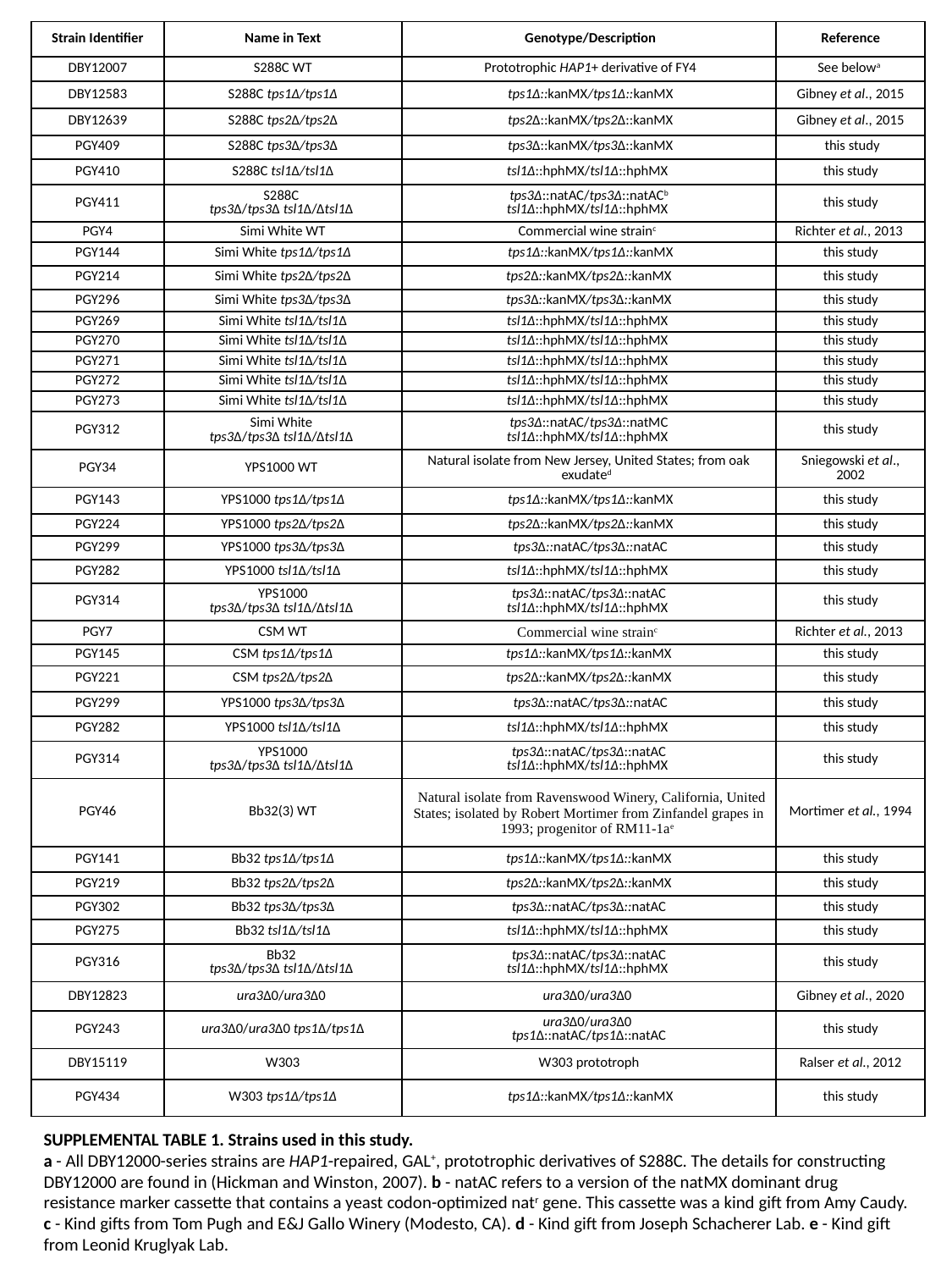

| Strain Identifier | Name in Text | Genotype/Description | Reference |
| --- | --- | --- | --- |
| DBY12007 | S288C WT | Prototrophic HAP1+ derivative of FY4 | See belowa |
| DBY12583 | S288C tps1Δ/tps1Δ | tps1Δ::kanMX/tps1Δ::kanMX | Gibney et al., 2015 |
| DBY12639 | S288C tps2Δ/tps2Δ | tps2Δ::kanMX/tps2Δ::kanMX | Gibney et al., 2015 |
| PGY409 | S288C tps3Δ/tps3Δ | tps3Δ::kanMX/tps3Δ::kanMX | this study |
| PGY410 | S288C tsl1Δ/tsl1Δ | tsl1Δ::hphMX/tsl1Δ::hphMX | this study |
| PGY411 | S288C tps3Δ/tps3Δ tsl1Δ/Δtsl1Δ | tps3Δ::natAC/tps3Δ::natACb tsl1Δ::hphMX/tsl1Δ::hphMX | this study |
| PGY4 | Simi White WT | Commercial wine strainc | Richter et al., 2013 |
| PGY144 | Simi White tps1Δ/tps1Δ | tps1Δ::kanMX/tps1Δ::kanMX | this study |
| PGY214 | Simi White tps2Δ/tps2Δ | tps2Δ::kanMX/tps2Δ::kanMX | this study |
| PGY296 | Simi White tps3Δ/tps3Δ | tps3Δ::kanMX/tps3Δ::kanMX | this study |
| PGY269 | Simi White tsl1Δ/tsl1Δ | tsl1Δ::hphMX/tsl1Δ::hphMX | this study |
| PGY270 | Simi White tsl1Δ/tsl1Δ | tsl1Δ::hphMX/tsl1Δ::hphMX | this study |
| PGY271 | Simi White tsl1Δ/tsl1Δ | tsl1Δ::hphMX/tsl1Δ::hphMX | this study |
| PGY272 | Simi White tsl1Δ/tsl1Δ | tsl1Δ::hphMX/tsl1Δ::hphMX | this study |
| PGY273 | Simi White tsl1Δ/tsl1Δ | tsl1Δ::hphMX/tsl1Δ::hphMX | this study |
| PGY312 | Simi White tps3Δ/tps3Δ tsl1Δ/Δtsl1Δ | tps3Δ::natAC/tps3Δ::natMC tsl1Δ::hphMX/tsl1Δ::hphMX | this study |
| PGY34 | YPS1000 WT | Natural isolate from New Jersey, United States; from oak exudated | Sniegowski et al., 2002 |
| PGY143 | YPS1000 tps1Δ/tps1Δ | tps1Δ::kanMX/tps1Δ::kanMX | this study |
| PGY224 | YPS1000 tps2Δ/tps2Δ | tps2Δ::kanMX/tps2Δ::kanMX | this study |
| PGY299 | YPS1000 tps3Δ/tps3Δ | tps3Δ::natAC/tps3Δ::natAC | this study |
| PGY282 | YPS1000 tsl1Δ/tsl1Δ | tsl1Δ::hphMX/tsl1Δ::hphMX | this study |
| PGY314 | YPS1000 tps3Δ/tps3Δ tsl1Δ/Δtsl1Δ | tps3Δ::natAC/tps3Δ::natAC tsl1Δ::hphMX/tsl1Δ::hphMX | this study |
| PGY7 | CSM WT | Commercial wine strainc | Richter et al., 2013 |
| PGY145 | CSM tps1Δ/tps1Δ | tps1Δ::kanMX/tps1Δ::kanMX | this study |
| PGY221 | CSM tps2Δ/tps2Δ | tps2Δ::kanMX/tps2Δ::kanMX | this study |
| PGY299 | YPS1000 tps3Δ/tps3Δ | tps3Δ::natAC/tps3Δ::natAC | this study |
| PGY282 | YPS1000 tsl1Δ/tsl1Δ | tsl1Δ::hphMX/tsl1Δ::hphMX | this study |
| PGY314 | YPS1000 tps3Δ/tps3Δ tsl1Δ/Δtsl1Δ | tps3Δ::natAC/tps3Δ::natAC tsl1Δ::hphMX/tsl1Δ::hphMX | this study |
| PGY46 | Bb32(3) WT | Natural isolate from Ravenswood Winery, California, United States; isolated by Robert Mortimer from Zinfandel grapes in 1993; progenitor of RM11-1ae | Mortimer et al., 1994 |
| PGY141 | Bb32 tps1Δ/tps1Δ | tps1Δ::kanMX/tps1Δ::kanMX | this study |
| PGY219 | Bb32 tps2Δ/tps2Δ | tps2Δ::kanMX/tps2Δ::kanMX | this study |
| PGY302 | Bb32 tps3Δ/tps3Δ | tps3Δ::natAC/tps3Δ::natAC | this study |
| PGY275 | Bb32 tsl1Δ/tsl1Δ | tsl1Δ::hphMX/tsl1Δ::hphMX | this study |
| PGY316 | Bb32 tps3Δ/tps3Δ tsl1Δ/Δtsl1Δ | tps3Δ::natAC/tps3Δ::natAC tsl1Δ::hphMX/tsl1Δ::hphMX | this study |
| DBY12823 | ura3Δ0/ura3Δ0 | ura3Δ0/ura3Δ0 | Gibney et al., 2020 |
| PGY243 | ura3Δ0/ura3Δ0 tps1Δ/tps1Δ | ura3Δ0/ura3Δ0 tps1Δ::natAC/tps1Δ::natAC | this study |
| DBY15119 | W303 | W303 prototroph | Ralser et al., 2012 |
| PGY434 | W303 tps1Δ/tps1Δ | tps1Δ::kanMX/tps1Δ::kanMX | this study |
SUPPLEMENTAL TABLE 1. Strains used in this study.
a - All DBY12000-series strains are HAP1-repaired, GAL+, prototrophic derivatives of S288C. The details for constructing DBY12000 are found in (Hickman and Winston, 2007). b - natAC refers to a version of the natMX dominant drug resistance marker cassette that contains a yeast codon-optimized natr gene. This cassette was a kind gift from Amy Caudy. c - Kind gifts from Tom Pugh and E&J Gallo Winery (Modesto, CA). d - Kind gift from Joseph Schacherer Lab. e - Kind gift from Leonid Kruglyak Lab.

## Slide 3
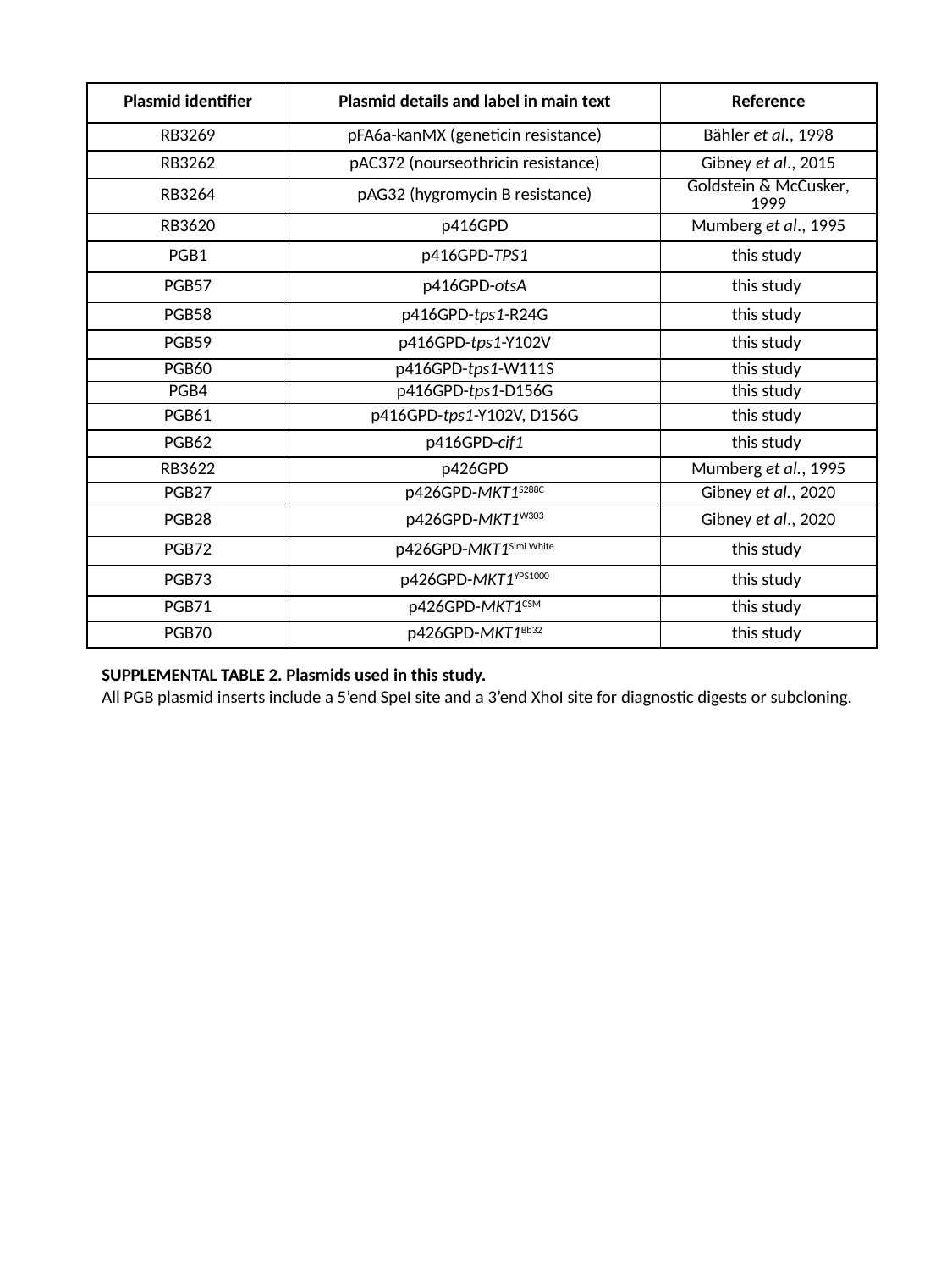

| Plasmid identifier | Plasmid details and label in main text | Reference |
| --- | --- | --- |
| RB3269 | pFA6a-kanMX (geneticin resistance) | Bähler et al., 1998 |
| RB3262 | pAC372 (nourseothricin resistance) | Gibney et al., 2015 |
| RB3264 | pAG32 (hygromycin B resistance) | Goldstein & McCusker, 1999 |
| RB3620 | p416GPD | Mumberg et al., 1995 |
| PGB1 | p416GPD-TPS1 | this study |
| PGB57 | p416GPD-otsA | this study |
| PGB58 | p416GPD-tps1-R24G | this study |
| PGB59 | p416GPD-tps1-Y102V | this study |
| PGB60 | p416GPD-tps1-W111S | this study |
| PGB4 | p416GPD-tps1-D156G | this study |
| PGB61 | p416GPD-tps1-Y102V, D156G | this study |
| PGB62 | p416GPD-cif1 | this study |
| RB3622 | p426GPD | Mumberg et al., 1995 |
| PGB27 | p426GPD-MKT1S288C | Gibney et al., 2020 |
| PGB28 | p426GPD-MKT1W303 | Gibney et al., 2020 |
| PGB72 | p426GPD-MKT1Simi White | this study |
| PGB73 | p426GPD-MKT1YPS1000 | this study |
| PGB71 | p426GPD-MKT1CSM | this study |
| PGB70 | p426GPD-MKT1Bb32 | this study |
SUPPLEMENTAL TABLE 2. Plasmids used in this study.
All PGB plasmid inserts include a 5’end SpeI site and a 3’end XhoI site for diagnostic digests or subcloning.

## Slide 4
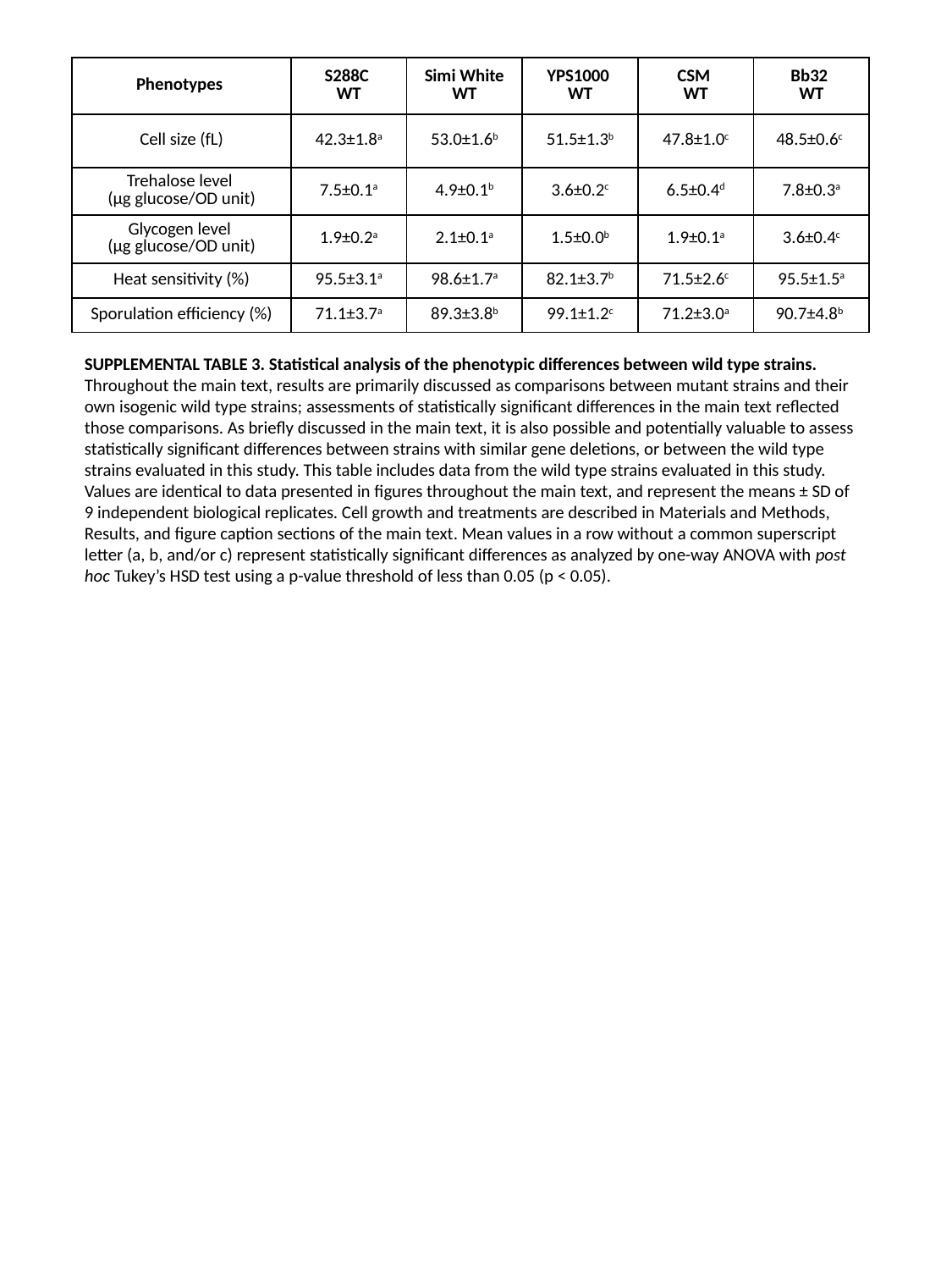

| Phenotypes | S288C WT | Simi White WT | YPS1000 WT | CSM WT | Bb32 WT |
| --- | --- | --- | --- | --- | --- |
| Cell size (fL) | 42.3±1.8a | 53.0±1.6b | 51.5±1.3b | 47.8±1.0c | 48.5±0.6c |
| Trehalose level (μg glucose/OD unit) | 7.5±0.1a | 4.9±0.1b | 3.6±0.2c | 6.5±0.4d | 7.8±0.3a |
| Glycogen level (μg glucose/OD unit) | 1.9±0.2a | 2.1±0.1a | 1.5±0.0b | 1.9±0.1a | 3.6±0.4c |
| Heat sensitivity (%) | 95.5±3.1a | 98.6±1.7a | 82.1±3.7b | 71.5±2.6c | 95.5±1.5a |
| Sporulation efficiency (%) | 71.1±3.7a | 89.3±3.8b | 99.1±1.2c | 71.2±3.0a | 90.7±4.8b |
SUPPLEMENTAL TABLE 3. Statistical analysis of the phenotypic differences between wild type strains.
Throughout the main text, results are primarily discussed as comparisons between mutant strains and their own isogenic wild type strains; assessments of statistically significant differences in the main text reflected those comparisons. As briefly discussed in the main text, it is also possible and potentially valuable to assess statistically significant differences between strains with similar gene deletions, or between the wild type strains evaluated in this study. This table includes data from the wild type strains evaluated in this study. Values are identical to data presented in figures throughout the main text, and represent the means ± SD of 9 independent biological replicates. Cell growth and treatments are described in Materials and Methods, Results, and figure caption sections of the main text. Mean values in a row without a common superscript letter (a, b, and/or c) represent statistically significant differences as analyzed by one-way ANOVA with post hoc Tukey’s HSD test using a p-value threshold of less than 0.05 (p < 0.05).

## Slide 5
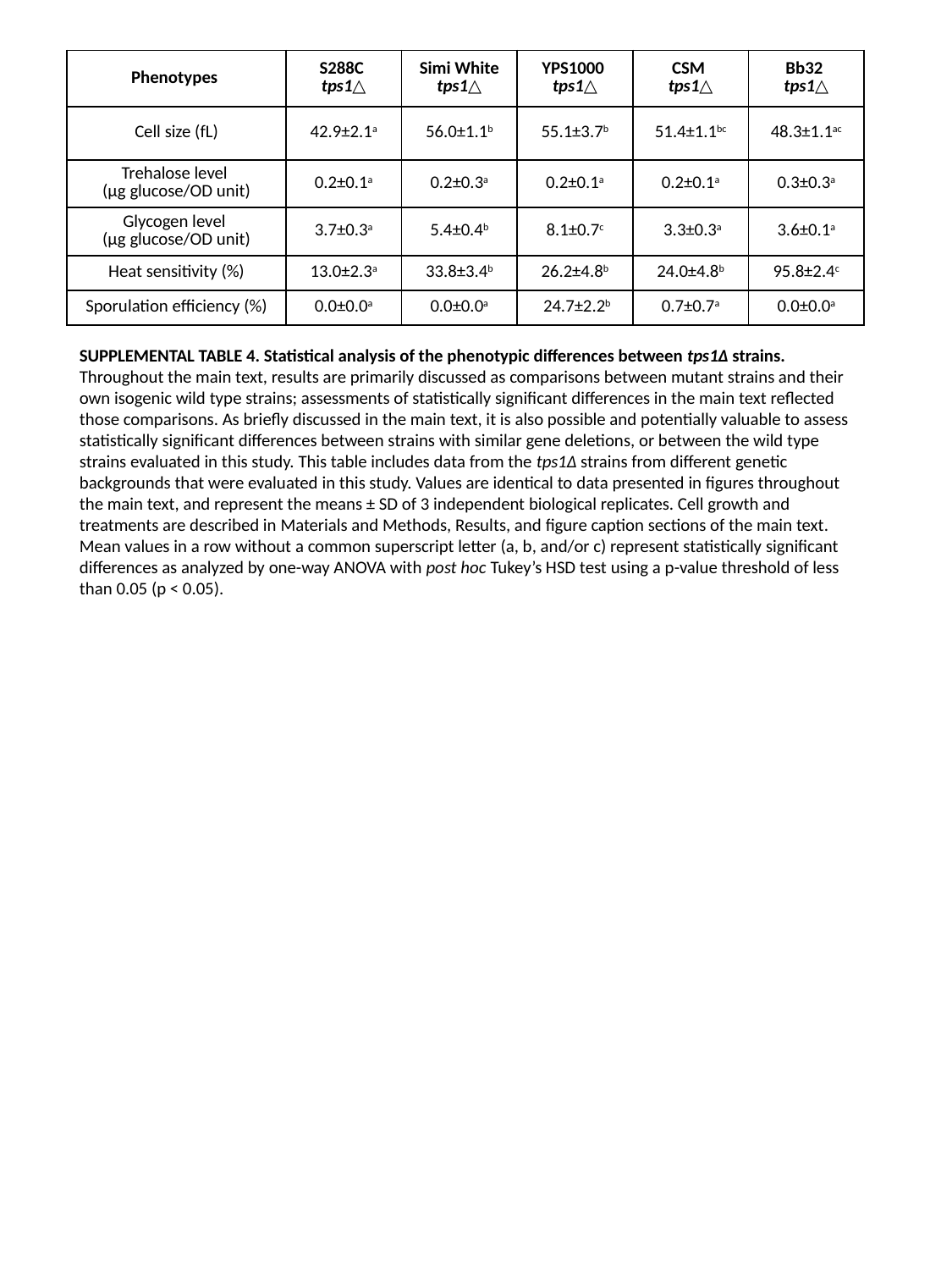

| Phenotypes | S288C tps1△ | Simi White tps1△ | YPS1000 tps1△ | CSM tps1△ | Bb32 tps1△ |
| --- | --- | --- | --- | --- | --- |
| Cell size (fL) | 42.9±2.1a | 56.0±1.1b | 55.1±3.7b | 51.4±1.1bc | 48.3±1.1ac |
| Trehalose level (μg glucose/OD unit) | 0.2±0.1a | 0.2±0.3a | 0.2±0.1a | 0.2±0.1a | 0.3±0.3a |
| Glycogen level (μg glucose/OD unit) | 3.7±0.3a | 5.4±0.4b | 8.1±0.7c | 3.3±0.3a | 3.6±0.1a |
| Heat sensitivity (%) | 13.0±2.3a | 33.8±3.4b | 26.2±4.8b | 24.0±4.8b | 95.8±2.4c |
| Sporulation efficiency (%) | 0.0±0.0a | 0.0±0.0a | 24.7±2.2b | 0.7±0.7a | 0.0±0.0a |
SUPPLEMENTAL TABLE 4. Statistical analysis of the phenotypic differences between tps1Δ strains.
Throughout the main text, results are primarily discussed as comparisons between mutant strains and their own isogenic wild type strains; assessments of statistically significant differences in the main text reflected those comparisons. As briefly discussed in the main text, it is also possible and potentially valuable to assess statistically significant differences between strains with similar gene deletions, or between the wild type strains evaluated in this study. This table includes data from the tps1Δ strains from different genetic backgrounds that were evaluated in this study. Values are identical to data presented in figures throughout the main text, and represent the means ± SD of 3 independent biological replicates. Cell growth and treatments are described in Materials and Methods, Results, and figure caption sections of the main text. Mean values in a row without a common superscript letter (a, b, and/or c) represent statistically significant differences as analyzed by one-way ANOVA with post hoc Tukey’s HSD test using a p-value threshold of less than 0.05 (p < 0.05).

## Slide 6
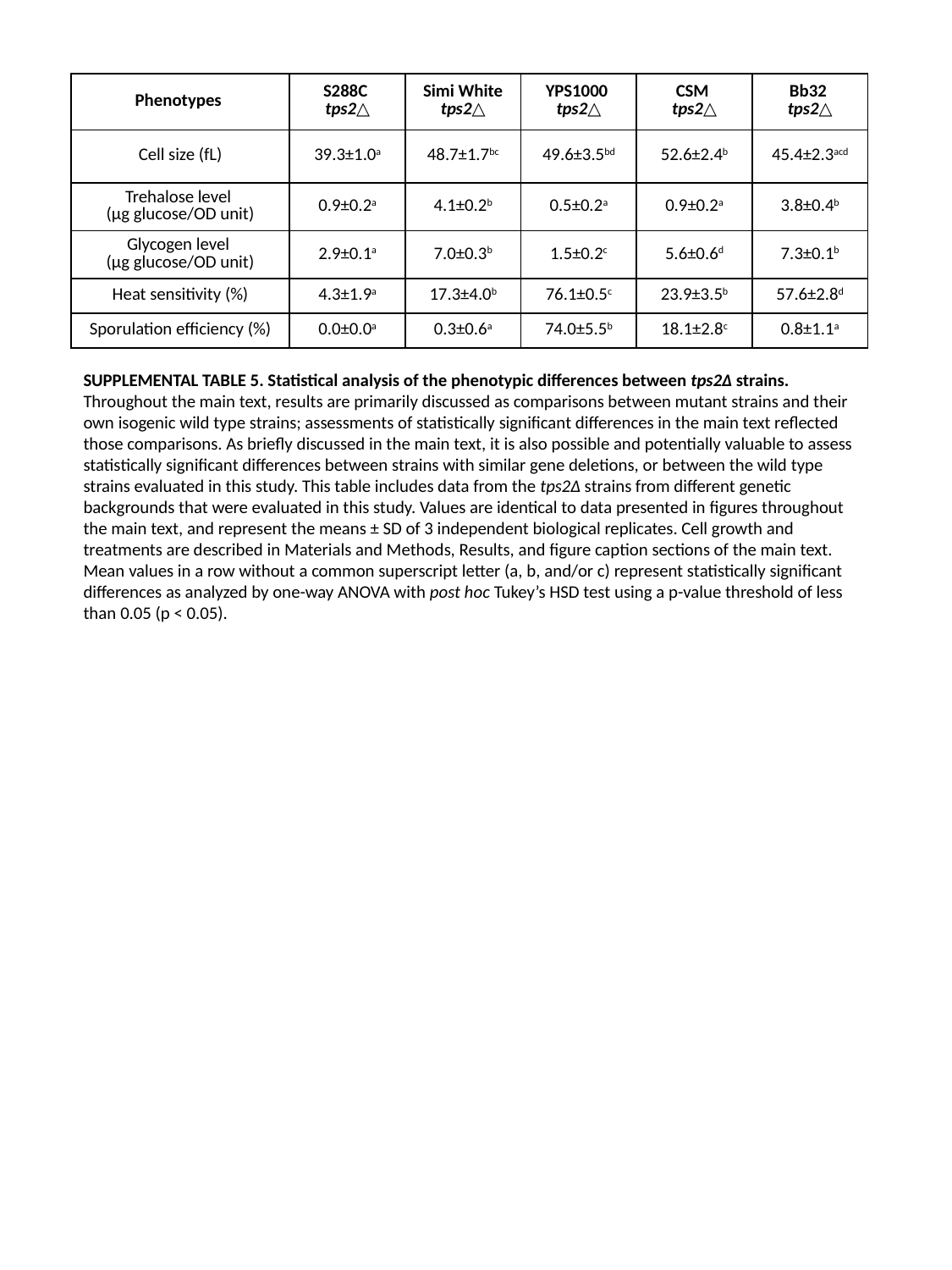

| Phenotypes | S288C tps2△ | Simi White tps2△ | YPS1000 tps2△ | CSM tps2△ | Bb32 tps2△ |
| --- | --- | --- | --- | --- | --- |
| Cell size (fL) | 39.3±1.0a | 48.7±1.7bc | 49.6±3.5bd | 52.6±2.4b | 45.4±2.3acd |
| Trehalose level (μg glucose/OD unit) | 0.9±0.2a | 4.1±0.2b | 0.5±0.2a | 0.9±0.2a | 3.8±0.4b |
| Glycogen level (μg glucose/OD unit) | 2.9±0.1a | 7.0±0.3b | 1.5±0.2c | 5.6±0.6d | 7.3±0.1b |
| Heat sensitivity (%) | 4.3±1.9a | 17.3±4.0b | 76.1±0.5c | 23.9±3.5b | 57.6±2.8d |
| Sporulation efficiency (%) | 0.0±0.0a | 0.3±0.6a | 74.0±5.5b | 18.1±2.8c | 0.8±1.1a |
SUPPLEMENTAL TABLE 5. Statistical analysis of the phenotypic differences between tps2Δ strains.
Throughout the main text, results are primarily discussed as comparisons between mutant strains and their own isogenic wild type strains; assessments of statistically significant differences in the main text reflected those comparisons. As briefly discussed in the main text, it is also possible and potentially valuable to assess statistically significant differences between strains with similar gene deletions, or between the wild type strains evaluated in this study. This table includes data from the tps2Δ strains from different genetic backgrounds that were evaluated in this study. Values are identical to data presented in figures throughout the main text, and represent the means ± SD of 3 independent biological replicates. Cell growth and treatments are described in Materials and Methods, Results, and figure caption sections of the main text. Mean values in a row without a common superscript letter (a, b, and/or c) represent statistically significant differences as analyzed by one-way ANOVA with post hoc Tukey’s HSD test using a p-value threshold of less than 0.05 (p < 0.05).

## Slide 7
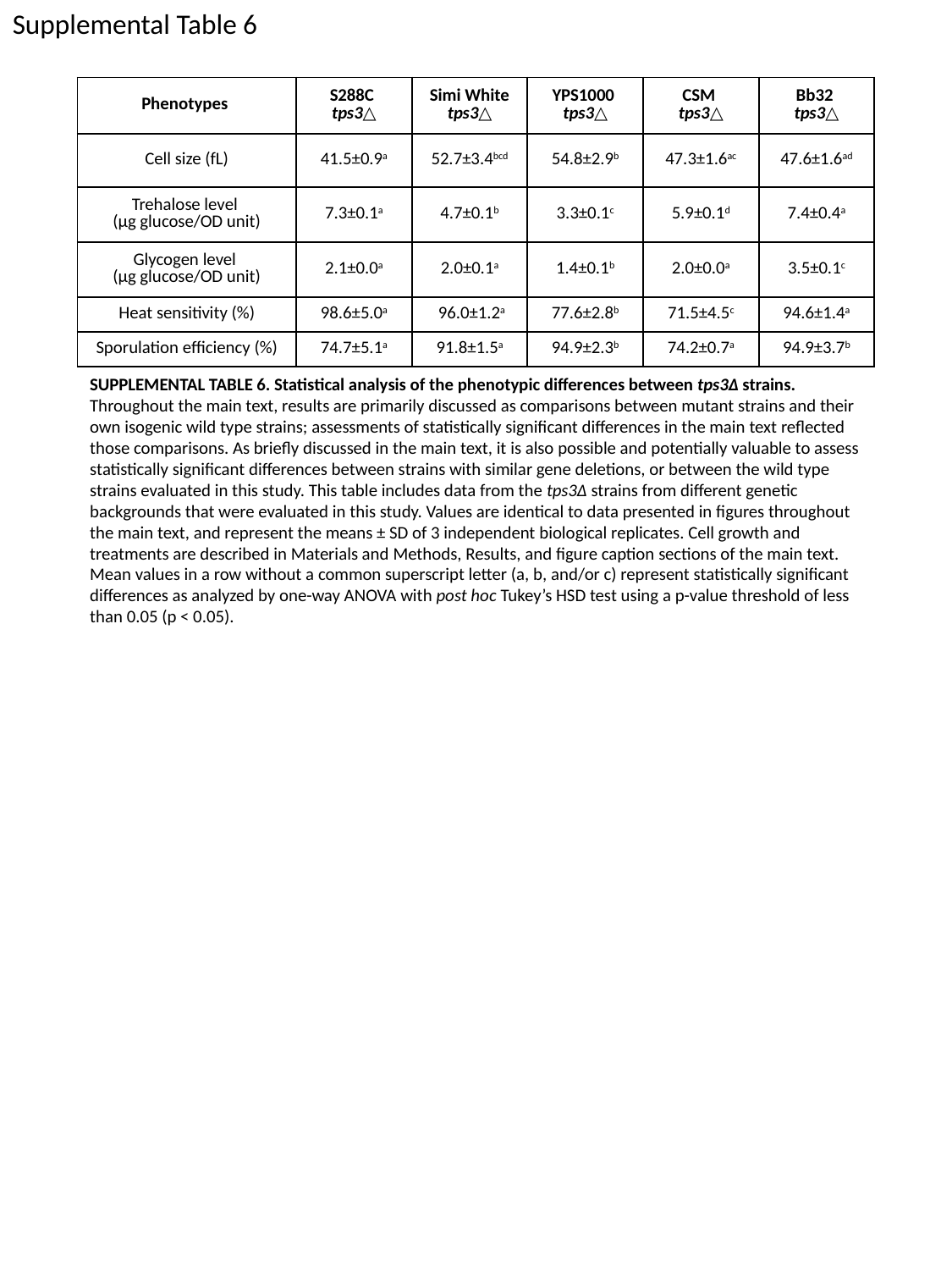

Supplemental Table 6
| Phenotypes | S288C tps3△ | Simi White tps3△ | YPS1000 tps3△ | CSM tps3△ | Bb32 tps3△ |
| --- | --- | --- | --- | --- | --- |
| Cell size (fL) | 41.5±0.9a | 52.7±3.4bcd | 54.8±2.9b | 47.3±1.6ac | 47.6±1.6ad |
| Trehalose level (μg glucose/OD unit) | 7.3±0.1a | 4.7±0.1b | 3.3±0.1c | 5.9±0.1d | 7.4±0.4a |
| Glycogen level (μg glucose/OD unit) | 2.1±0.0a | 2.0±0.1a | 1.4±0.1b | 2.0±0.0a | 3.5±0.1c |
| Heat sensitivity (%) | 98.6±5.0a | 96.0±1.2a | 77.6±2.8b | 71.5±4.5c | 94.6±1.4a |
| Sporulation efficiency (%) | 74.7±5.1a | 91.8±1.5a | 94.9±2.3b | 74.2±0.7a | 94.9±3.7b |
SUPPLEMENTAL TABLE 6. Statistical analysis of the phenotypic differences between tps3Δ strains.
Throughout the main text, results are primarily discussed as comparisons between mutant strains and their own isogenic wild type strains; assessments of statistically significant differences in the main text reflected those comparisons. As briefly discussed in the main text, it is also possible and potentially valuable to assess statistically significant differences between strains with similar gene deletions, or between the wild type strains evaluated in this study. This table includes data from the tps3Δ strains from different genetic backgrounds that were evaluated in this study. Values are identical to data presented in figures throughout the main text, and represent the means ± SD of 3 independent biological replicates. Cell growth and treatments are described in Materials and Methods, Results, and figure caption sections of the main text. Mean values in a row without a common superscript letter (a, b, and/or c) represent statistically significant differences as analyzed by one-way ANOVA with post hoc Tukey’s HSD test using a p-value threshold of less than 0.05 (p < 0.05).

## Slide 8
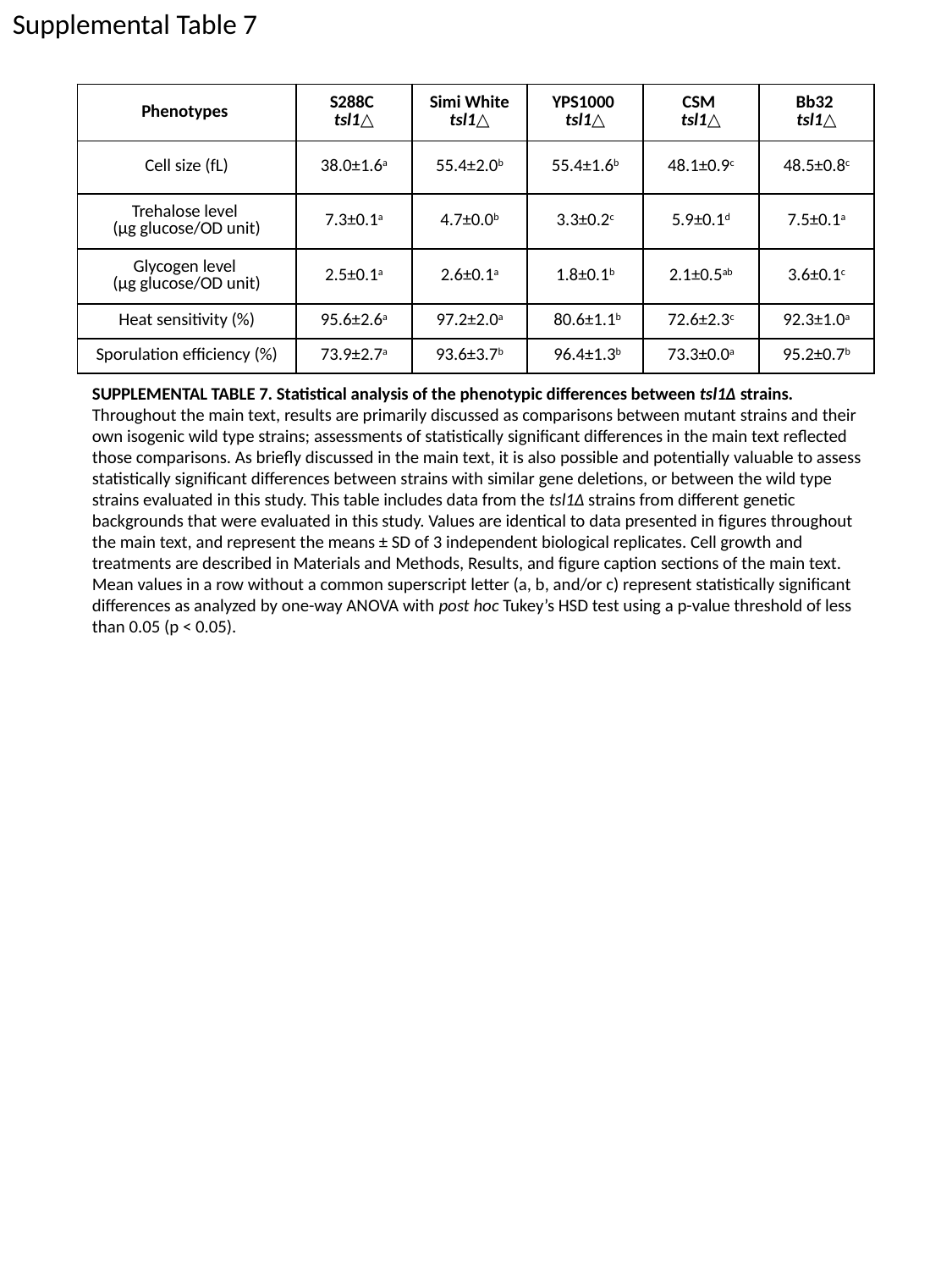

Supplemental Table 7
| Phenotypes | S288C tsl1△ | Simi White tsl1△ | YPS1000 tsl1△ | CSM tsl1△ | Bb32 tsl1△ |
| --- | --- | --- | --- | --- | --- |
| Cell size (fL) | 38.0±1.6a | 55.4±2.0b | 55.4±1.6b | 48.1±0.9c | 48.5±0.8c |
| Trehalose level (μg glucose/OD unit) | 7.3±0.1a | 4.7±0.0b | 3.3±0.2c | 5.9±0.1d | 7.5±0.1a |
| Glycogen level (μg glucose/OD unit) | 2.5±0.1a | 2.6±0.1a | 1.8±0.1b | 2.1±0.5ab | 3.6±0.1c |
| Heat sensitivity (%) | 95.6±2.6a | 97.2±2.0a | 80.6±1.1b | 72.6±2.3c | 92.3±1.0a |
| Sporulation efficiency (%) | 73.9±2.7a | 93.6±3.7b | 96.4±1.3b | 73.3±0.0a | 95.2±0.7b |
SUPPLEMENTAL TABLE 7. Statistical analysis of the phenotypic differences between tsl1Δ strains.
Throughout the main text, results are primarily discussed as comparisons between mutant strains and their own isogenic wild type strains; assessments of statistically significant differences in the main text reflected those comparisons. As briefly discussed in the main text, it is also possible and potentially valuable to assess statistically significant differences between strains with similar gene deletions, or between the wild type strains evaluated in this study. This table includes data from the tsl1Δ strains from different genetic backgrounds that were evaluated in this study. Values are identical to data presented in figures throughout the main text, and represent the means ± SD of 3 independent biological replicates. Cell growth and treatments are described in Materials and Methods, Results, and figure caption sections of the main text. Mean values in a row without a common superscript letter (a, b, and/or c) represent statistically significant differences as analyzed by one-way ANOVA with post hoc Tukey’s HSD test using a p-value threshold of less than 0.05 (p < 0.05).

## Slide 9
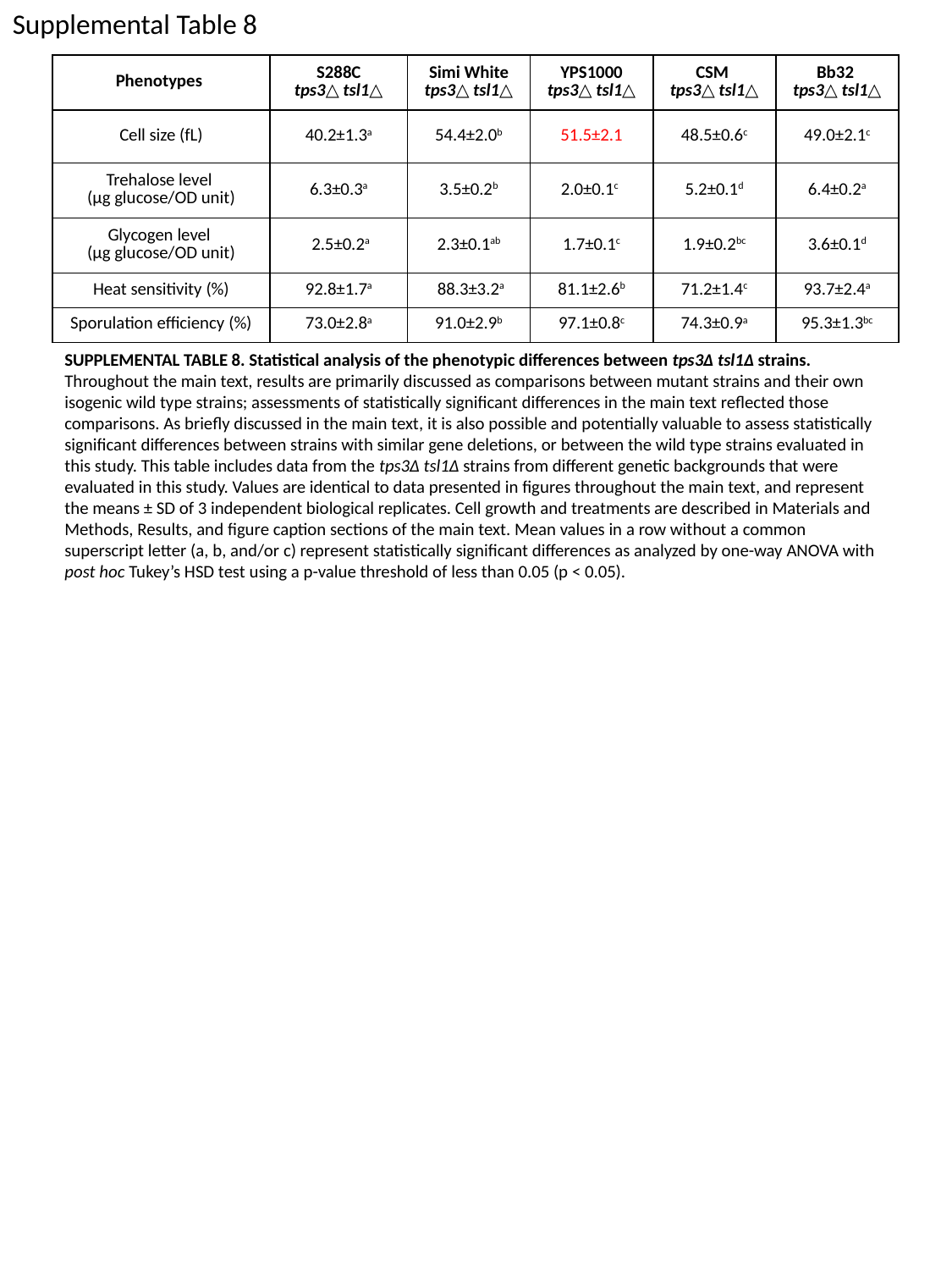

Supplemental Table 8
| Phenotypes | S288C tps3△ tsl1△ | Simi White tps3△ tsl1△ | YPS1000 tps3△ tsl1△ | CSM tps3△ tsl1△ | Bb32 tps3△ tsl1△ |
| --- | --- | --- | --- | --- | --- |
| Cell size (fL) | 40.2±1.3a | 54.4±2.0b | 51.5±2.1 | 48.5±0.6c | 49.0±2.1c |
| Trehalose level (μg glucose/OD unit) | 6.3±0.3a | 3.5±0.2b | 2.0±0.1c | 5.2±0.1d | 6.4±0.2a |
| Glycogen level (μg glucose/OD unit) | 2.5±0.2a | 2.3±0.1ab | 1.7±0.1c | 1.9±0.2bc | 3.6±0.1d |
| Heat sensitivity (%) | 92.8±1.7a | 88.3±3.2a | 81.1±2.6b | 71.2±1.4c | 93.7±2.4a |
| Sporulation efficiency (%) | 73.0±2.8a | 91.0±2.9b | 97.1±0.8c | 74.3±0.9a | 95.3±1.3bc |
SUPPLEMENTAL TABLE 8. Statistical analysis of the phenotypic differences between tps3Δ tsl1Δ strains.
Throughout the main text, results are primarily discussed as comparisons between mutant strains and their own isogenic wild type strains; assessments of statistically significant differences in the main text reflected those comparisons. As briefly discussed in the main text, it is also possible and potentially valuable to assess statistically significant differences between strains with similar gene deletions, or between the wild type strains evaluated in this study. This table includes data from the tps3Δ tsl1Δ strains from different genetic backgrounds that were evaluated in this study. Values are identical to data presented in figures throughout the main text, and represent the means ± SD of 3 independent biological replicates. Cell growth and treatments are described in Materials and Methods, Results, and figure caption sections of the main text. Mean values in a row without a common superscript letter (a, b, and/or c) represent statistically significant differences as analyzed by one-way ANOVA with post hoc Tukey’s HSD test using a p-value threshold of less than 0.05 (p < 0.05).

## Slide 10
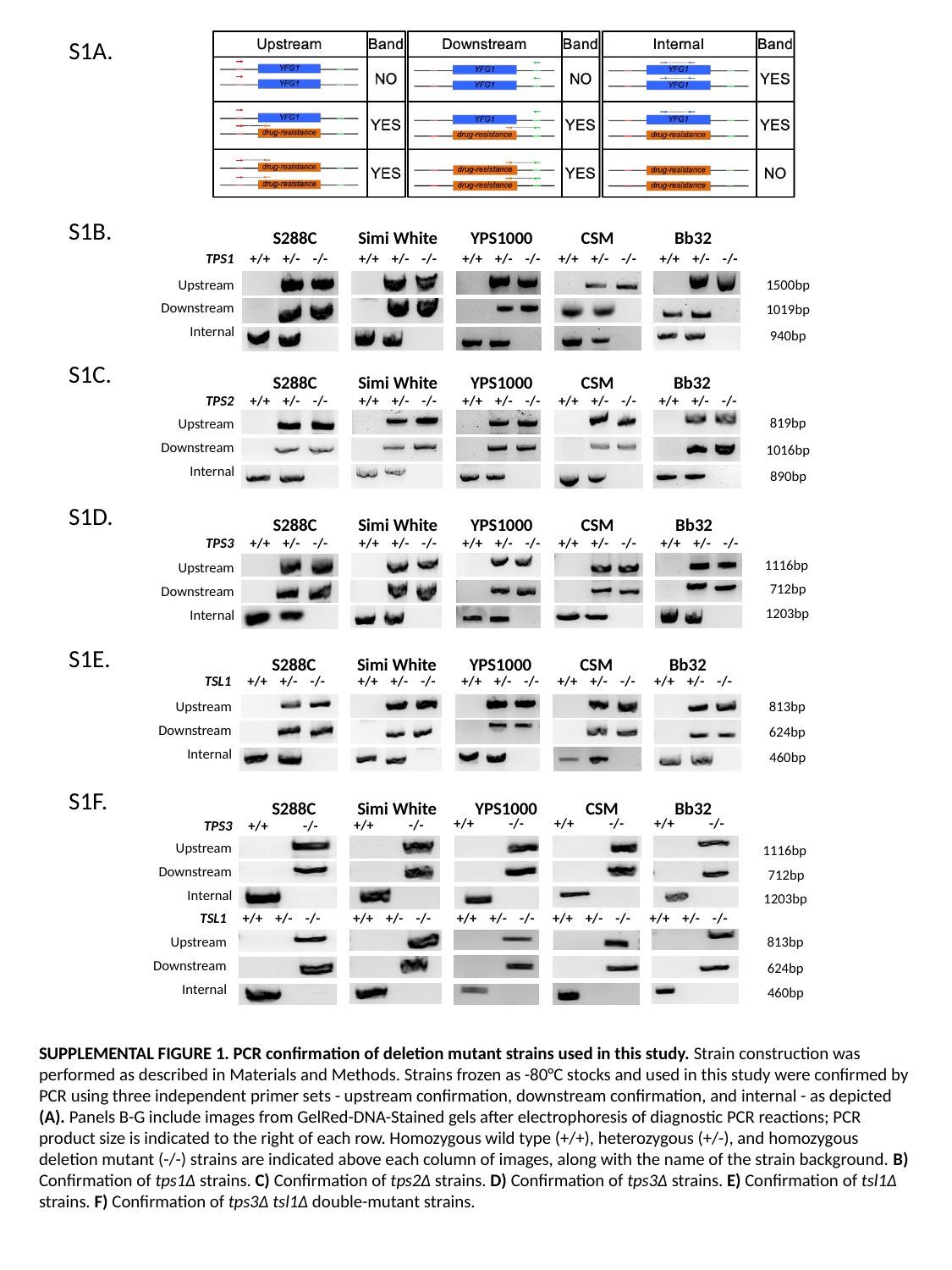

S1A.
S1B.
S288C
Simi White
YPS1000
CSM
Bb32
TPS1 +/+ +/- -/-
+/+ +/- -/-
+/+ +/- -/-
+/+ +/- -/-
+/+ +/- -/-
Upstream
1500bp
Downstream
1019bp
Internal
940bp
S1C.
S288C
Simi White
YPS1000
CSM
Bb32
TPS2 +/+ +/- -/-
+/+ +/- -/-
+/+ +/- -/-
+/+ +/- -/-
+/+ +/- -/-
819bp
Upstream
Downstream
1016bp
Internal
890bp
S1D.
S288C
Simi White
YPS1000
CSM
Bb32
TPS3 +/+ +/- -/-
+/+ +/- -/-
+/+ +/- -/-
+/+ +/- -/-
+/+ +/- -/-
1116bp
Upstream
712bp
Downstream
1203bp
Internal
S1E.
S288C
Simi White
YPS1000
CSM
Bb32
TSL1 +/+ +/- -/-
+/+ +/- -/-
+/+ +/- -/-
+/+ +/- -/-
+/+ +/- -/-
813bp
Upstream
Downstream
624bp
Internal
460bp
S1F.
S288C
Simi White
YPS1000
CSM
Bb32
+/+ -/-
+/+ -/-
+/+ -/-
+/+ -/-
TPS3 +/+ -/-
Upstream
1116bp
Downstream
712bp
Internal
1203bp
TSL1 +/+ +/- -/-
+/+ +/- -/-
+/+ +/- -/-
+/+ +/- -/-
+/+ +/- -/-
813bp
Upstream
Downstream
624bp
Internal
460bp
SUPPLEMENTAL FIGURE 1. PCR confirmation of deletion mutant strains used in this study. Strain construction was performed as described in Materials and Methods. Strains frozen as -80°C stocks and used in this study were confirmed by PCR using three independent primer sets - upstream confirmation, downstream confirmation, and internal - as depicted (A). Panels B-G include images from GelRed-DNA-Stained gels after electrophoresis of diagnostic PCR reactions; PCR product size is indicated to the right of each row. Homozygous wild type (+/+), heterozygous (+/-), and homozygous deletion mutant (-/-) strains are indicated above each column of images, along with the name of the strain background. B) Confirmation of tps1Δ strains. C) Confirmation of tps2Δ strains. D) Confirmation of tps3Δ strains. E) Confirmation of tsl1Δ strains. F) Confirmation of tps3Δ tsl1Δ double-mutant strains.

## Slide 11
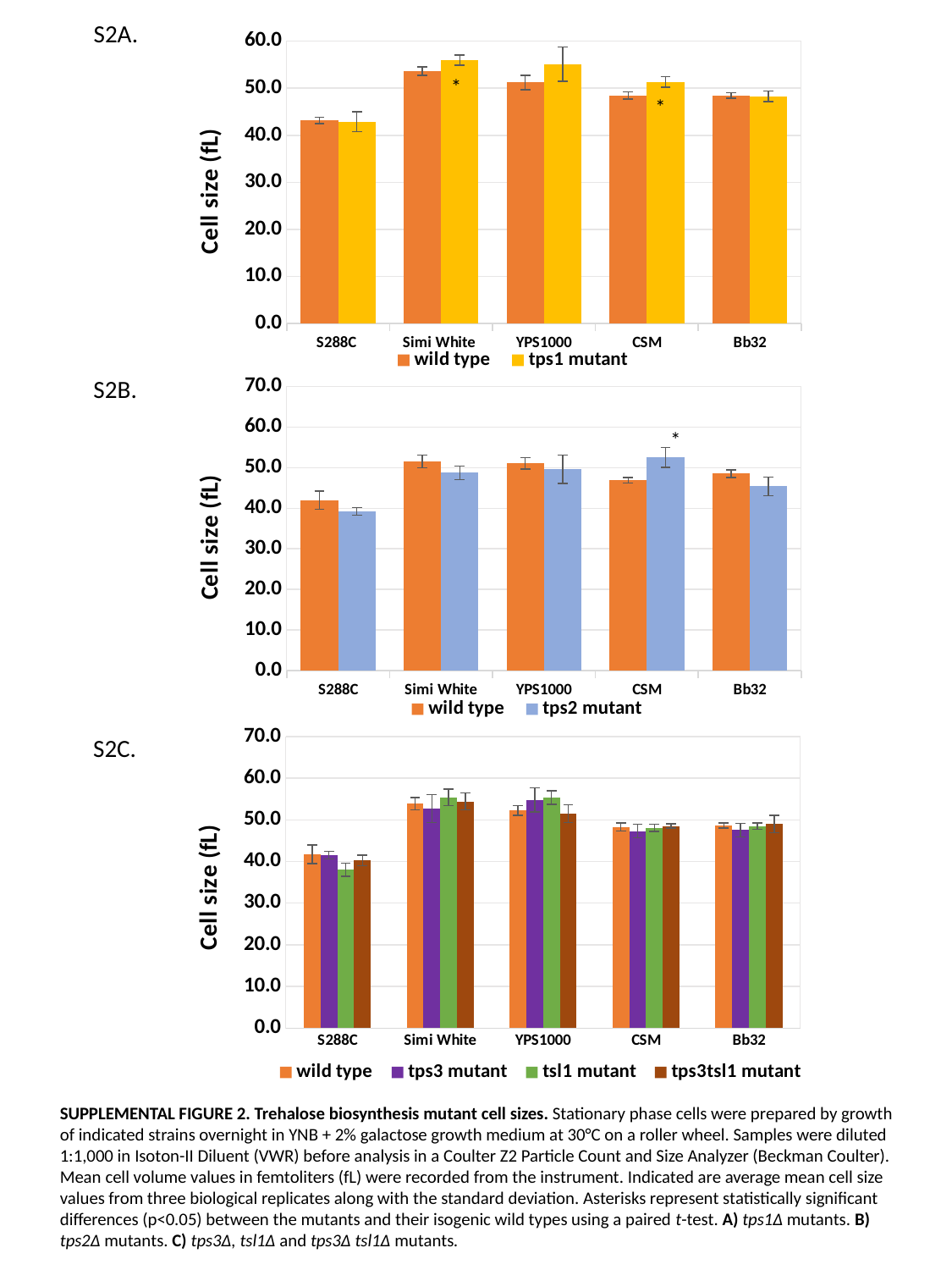

S2A.
### Chart
| Category | | |
|---|---|---|
| S288C | 43.14506666666653 | 42.91673333333333 |
| Simi White | 53.6542 | 55.9876 |
| YPS1000 | 51.21360000000001 | 55.11666666666648 |
| CSM | 48.44600000000001 | 51.35733333333332 |
| Bb32 | 48.4352 | 48.30260000000001 |*
*
S2B.
### Chart
| Category | | |
|---|---|---|
| S288C | 41.95873333333333 | 39.2505 |
| Simi White | 51.55756666666645 | 48.74140000000001 |
| YPS1000 | 51.05583333333333 | 49.60206666666653 |
| CSM | 46.8457 | 52.55166666666641 |
| Bb32 | 48.5467 | 45.408 |*
### Chart
| Category | | | | |
|---|---|---|---|---|
| S288C | 41.71 | 41.5474 | 38.01706666666645 | 40.2403 |
| Simi White | 53.8566 | 52.69920000000001 | 55.392 | 54.43136666666648 |
| YPS1000 | 52.22933333333333 | 54.82523333333334 | 55.37886666666644 | 51.497 |
| CSM | 48.28693333333333 | 47.3132 | 48.05353333333333 | 48.5364 |
| Bb32 | 48.65166666666644 | 47.56 | 48.48886666666645 | 48.99836666666655 |S2C.
SUPPLEMENTAL FIGURE 2. Trehalose biosynthesis mutant cell sizes. Stationary phase cells were prepared by growth of indicated strains overnight in YNB + 2% galactose growth medium at 30°C on a roller wheel. Samples were diluted 1:1,000 in Isoton-II Diluent (VWR) before analysis in a Coulter Z2 Particle Count and Size Analyzer (Beckman Coulter). Mean cell volume values in femtoliters (fL) were recorded from the instrument. Indicated are average mean cell size values from three biological replicates along with the standard deviation. Asterisks represent statistically significant differences (p<0.05) between the mutants and their isogenic wild types using a paired t-test. A) tps1Δ mutants. B) tps2Δ mutants. C) tps3Δ, tsl1Δ and tps3Δ tsl1Δ mutants.

## Slide 12
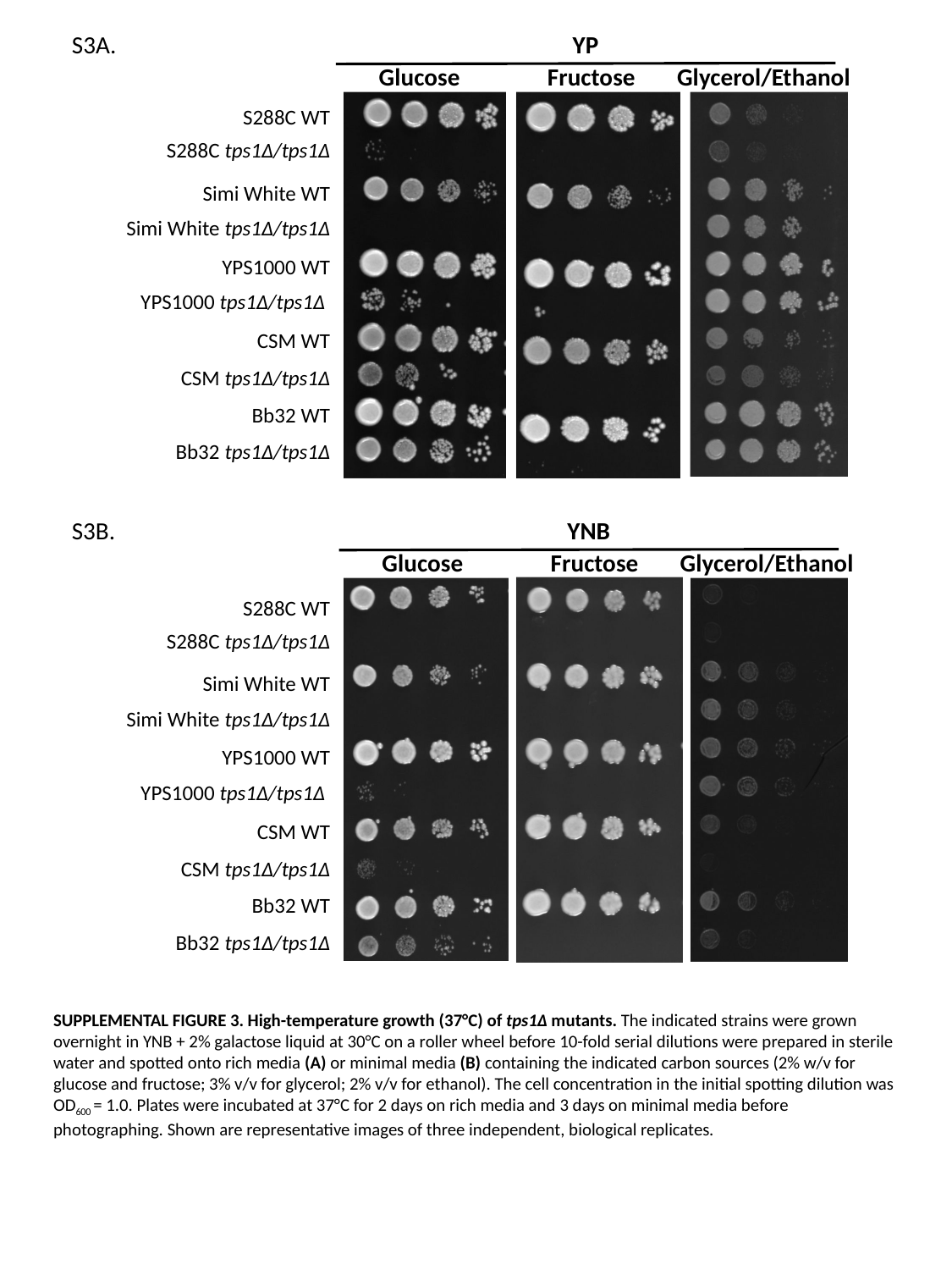

S3A.
YP
Glucose
Fructose
Glycerol/Ethanol
S288C WT
S288C tps1Δ/tps1Δ
Simi White WT
Simi White tps1Δ/tps1Δ
YPS1000 WT
YPS1000 tps1Δ/tps1Δ
CSM WT
CSM tps1Δ/tps1Δ
Bb32 WT
Bb32 tps1Δ/tps1Δ
S3B.
YNB
Glucose
Fructose
Glycerol/Ethanol
S288C WT
S288C tps1Δ/tps1Δ
Simi White WT
Simi White tps1Δ/tps1Δ
YPS1000 WT
YPS1000 tps1Δ/tps1Δ
CSM WT
CSM tps1Δ/tps1Δ
Bb32 WT
Bb32 tps1Δ/tps1Δ
SUPPLEMENTAL FIGURE 3. High-temperature growth (37°C) of tps1Δ mutants. The indicated strains were grown overnight in YNB + 2% galactose liquid at 30°C on a roller wheel before 10-fold serial dilutions were prepared in sterile water and spotted onto rich media (A) or minimal media (B) containing the indicated carbon sources (2% w/v for glucose and fructose; 3% v/v for glycerol; 2% v/v for ethanol). The cell concentration in the initial spotting dilution was OD600 = 1.0. Plates were incubated at 37°C for 2 days on rich media and 3 days on minimal media before photographing. Shown are representative images of three independent, biological replicates.

## Slide 13
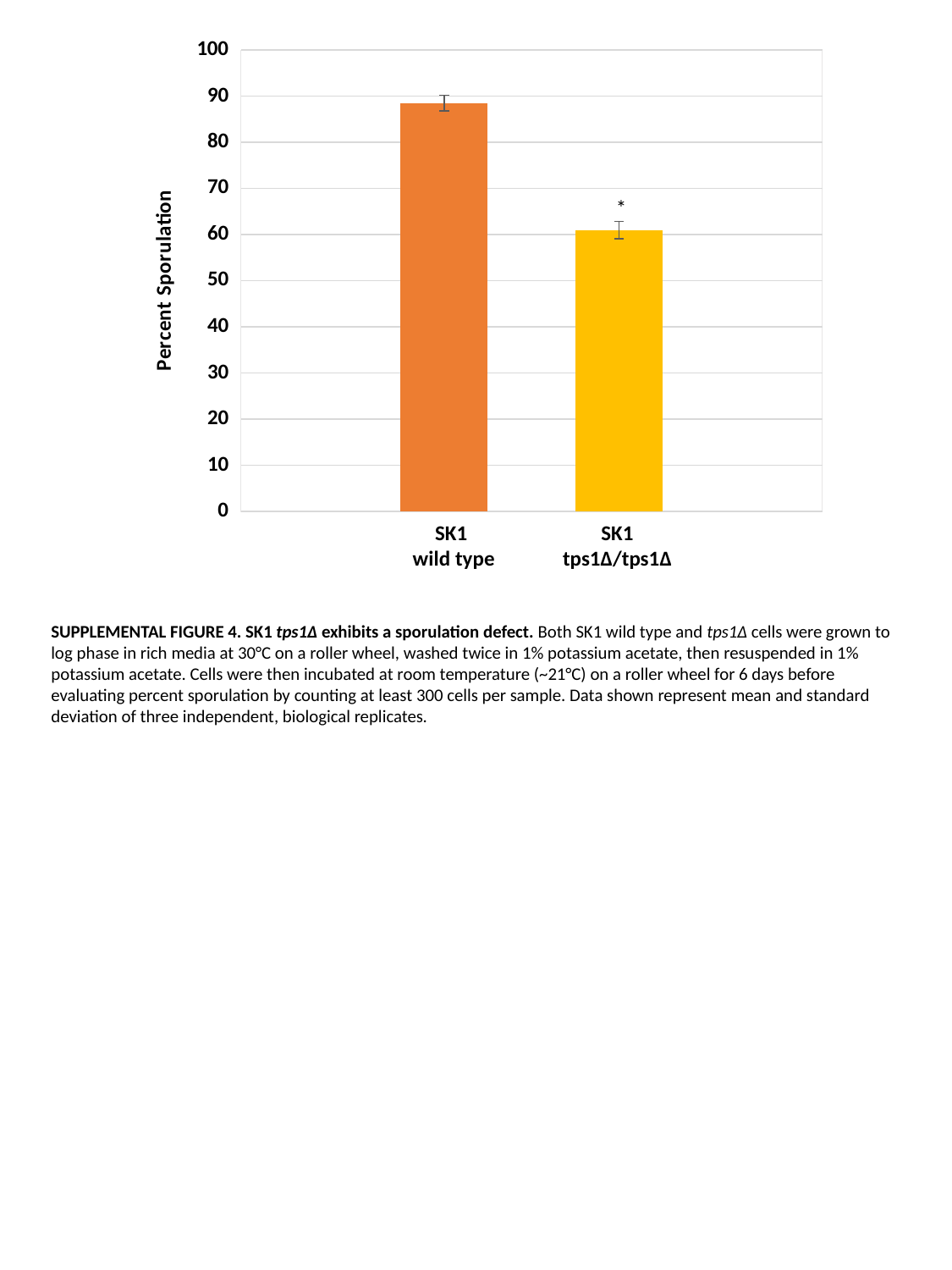

### Chart
| Category | | |
|---|---|---|*
SK1
wild type
SK1
tps1Δ/tps1Δ
SUPPLEMENTAL FIGURE 4. SK1 tps1Δ exhibits a sporulation defect. Both SK1 wild type and tps1Δ cells were grown to log phase in rich media at 30°C on a roller wheel, washed twice in 1% potassium acetate, then resuspended in 1% potassium acetate. Cells were then incubated at room temperature (~21°C) on a roller wheel for 6 days before evaluating percent sporulation by counting at least 300 cells per sample. Data shown represent mean and standard deviation of three independent, biological replicates.

## Slide 14
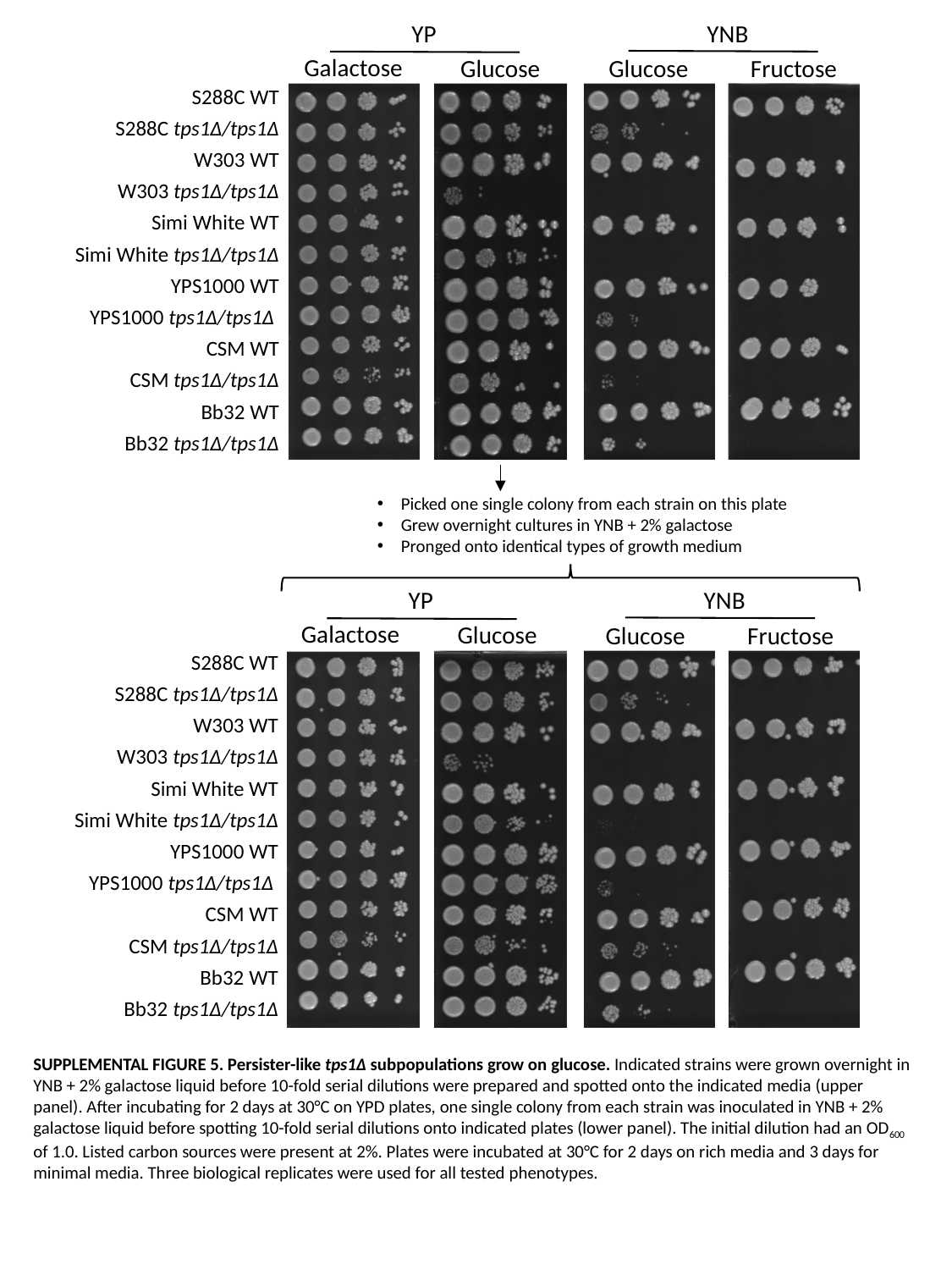

YP
YNB
Galactose
Glucose
Glucose
Fructose
S288C WT
S288C tps1Δ/tps1Δ
W303 WT
W303 tps1Δ/tps1Δ
Simi White WT
Simi White tps1Δ/tps1Δ
YPS1000 WT
YPS1000 tps1Δ/tps1Δ
CSM WT
CSM tps1Δ/tps1Δ
Bb32 WT
Bb32 tps1Δ/tps1Δ
Picked one single colony from each strain on this plate
Grew overnight cultures in YNB + 2% galactose
Pronged onto identical types of growth medium
YP
YNB
Galactose
Glucose
Glucose
Fructose
S288C WT
S288C tps1Δ/tps1Δ
W303 WT
W303 tps1Δ/tps1Δ
Simi White WT
Simi White tps1Δ/tps1Δ
YPS1000 WT
YPS1000 tps1Δ/tps1Δ
CSM WT
CSM tps1Δ/tps1Δ
Bb32 WT
Bb32 tps1Δ/tps1Δ
SUPPLEMENTAL FIGURE 5. Persister-like tps1Δ subpopulations grow on glucose. Indicated strains were grown overnight in YNB + 2% galactose liquid before 10-fold serial dilutions were prepared and spotted onto the indicated media (upper panel). After incubating for 2 days at 30°C on YPD plates, one single colony from each strain was inoculated in YNB + 2% galactose liquid before spotting 10-fold serial dilutions onto indicated plates (lower panel). The initial dilution had an OD600 of 1.0. Listed carbon sources were present at 2%. Plates were incubated at 30°C for 2 days on rich media and 3 days for minimal media. Three biological replicates were used for all tested phenotypes.

## Slide 15
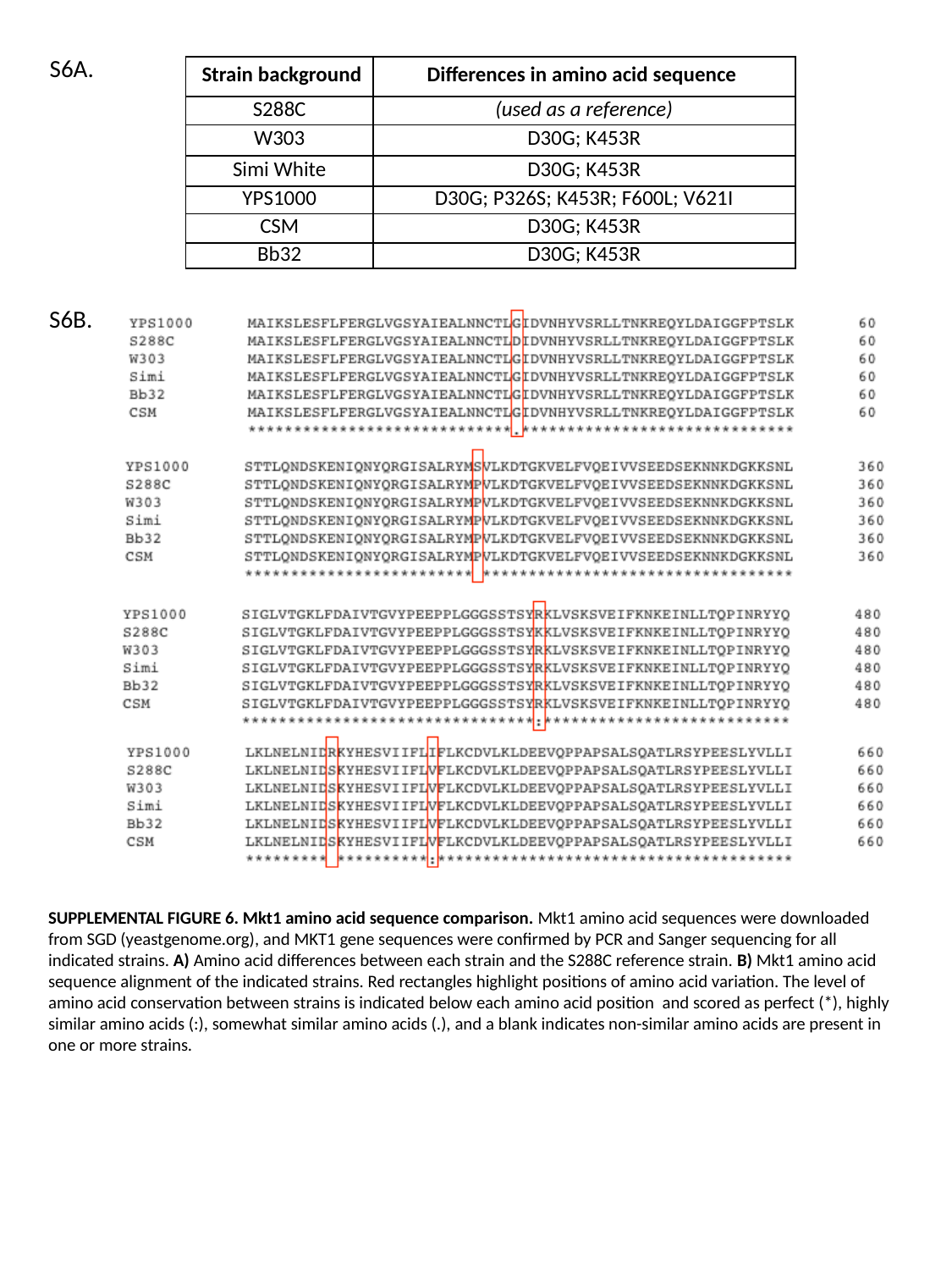

S6A.
| Strain background | Differences in amino acid sequence |
| --- | --- |
| S288C | (used as a reference) |
| W303 | D30G; K453R |
| Simi White | D30G; K453R |
| YPS1000 | D30G; P326S; K453R; F600L; V621I |
| CSM | D30G; K453R |
| Bb32 | D30G; K453R |
S6B.
SUPPLEMENTAL FIGURE 6. Mkt1 amino acid sequence comparison. Mkt1 amino acid sequences were downloaded from SGD (yeastgenome.org), and MKT1 gene sequences were confirmed by PCR and Sanger sequencing for all indicated strains. A) Amino acid differences between each strain and the S288C reference strain. B) Mkt1 amino acid sequence alignment of the indicated strains. Red rectangles highlight positions of amino acid variation. The level of amino acid conservation between strains is indicated below each amino acid position and scored as perfect (*), highly similar amino acids (:), somewhat similar amino acids (.), and a blank indicates non-similar amino acids are present in one or more strains.

## Slide 16
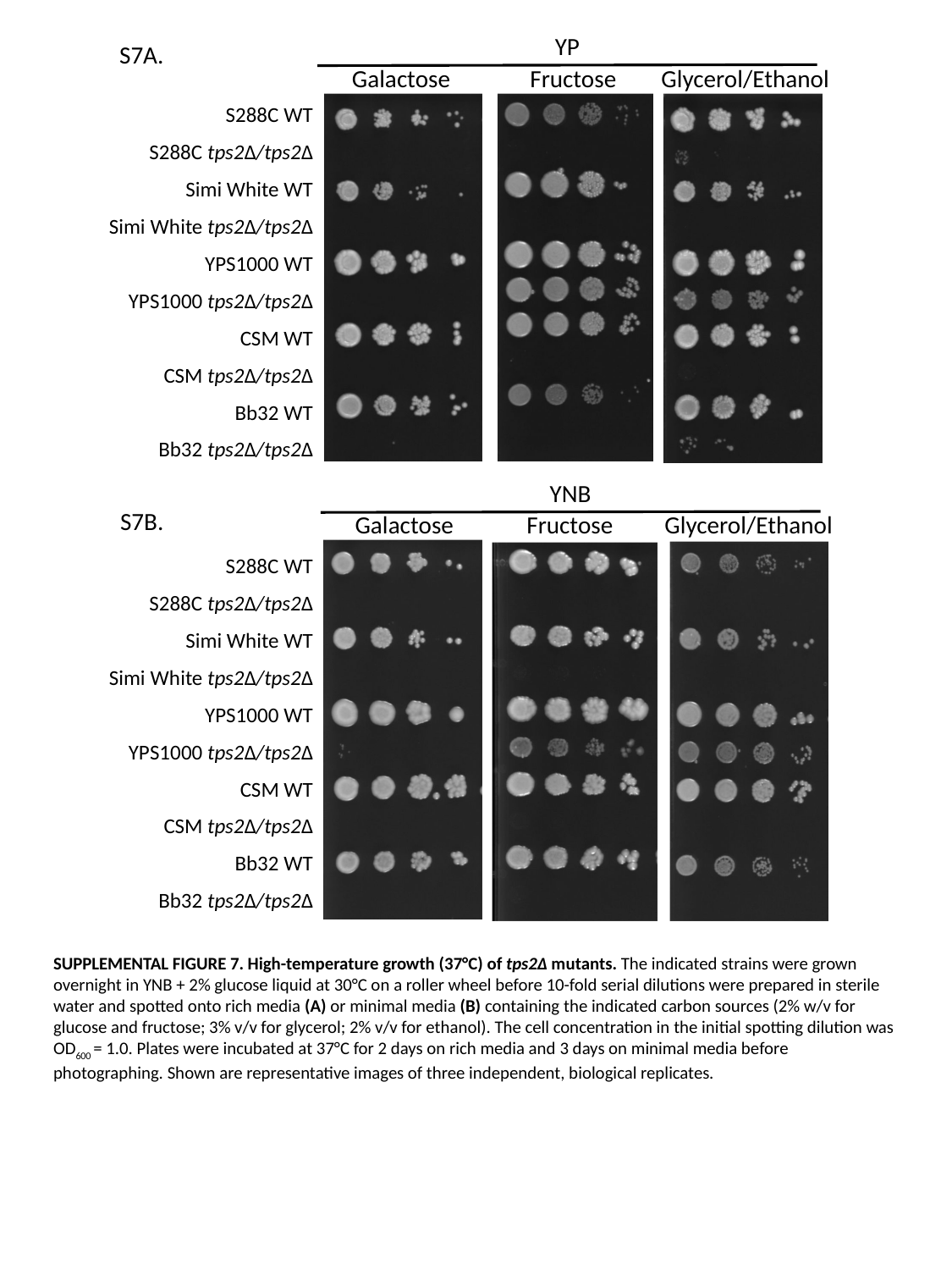

YP
S7A.
Galactose
Fructose
Glycerol/Ethanol
S288C WT
S288C tps2Δ/tps2Δ
Simi White WT
Simi White tps2Δ/tps2Δ
YPS1000 WT
YPS1000 tps2Δ/tps2Δ
CSM WT
CSM tps2Δ/tps2Δ
Bb32 WT
Bb32 tps2Δ/tps2Δ
YNB
S7B.
Galactose
Fructose
Glycerol/Ethanol
S288C WT
S288C tps2Δ/tps2Δ
Simi White WT
Simi White tps2Δ/tps2Δ
YPS1000 WT
YPS1000 tps2Δ/tps2Δ
CSM WT
CSM tps2Δ/tps2Δ
Bb32 WT
Bb32 tps2Δ/tps2Δ
SUPPLEMENTAL FIGURE 7. High-temperature growth (37°C) of tps2Δ mutants. The indicated strains were grown overnight in YNB + 2% glucose liquid at 30°C on a roller wheel before 10-fold serial dilutions were prepared in sterile water and spotted onto rich media (A) or minimal media (B) containing the indicated carbon sources (2% w/v for glucose and fructose; 3% v/v for glycerol; 2% v/v for ethanol). The cell concentration in the initial spotting dilution was OD600 = 1.0. Plates were incubated at 37°C for 2 days on rich media and 3 days on minimal media before photographing. Shown are representative images of three independent, biological replicates.

## Slide 17
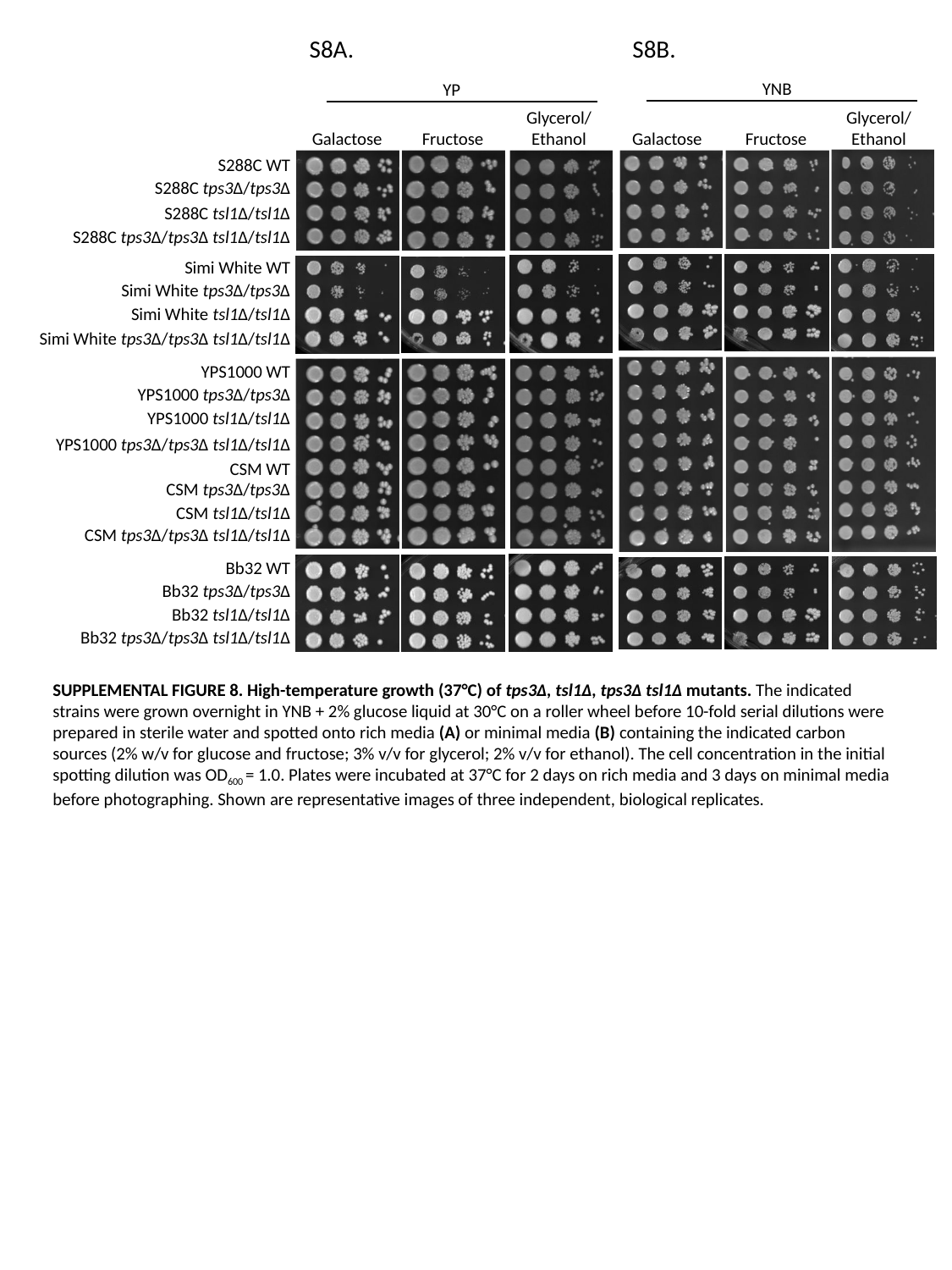

S8A.
S8B.
YNB
YP
Glycerol/
Ethanol
Glycerol/
Ethanol
Galactose
Fructose
Galactose
Fructose
S288C WT
S288C tps3Δ/tps3Δ
S288C tsl1Δ/tsl1Δ
S288C tps3Δ/tps3Δ tsl1Δ/tsl1Δ
Simi White WT
Simi White tps3Δ/tps3Δ
Simi White tsl1Δ/tsl1Δ
Simi White tps3Δ/tps3Δ tsl1Δ/tsl1Δ
YPS1000 WT
YPS1000 tps3Δ/tps3Δ
YPS1000 tsl1Δ/tsl1Δ
YPS1000 tps3Δ/tps3Δ tsl1Δ/tsl1Δ
CSM WT
CSM tps3Δ/tps3Δ
CSM tsl1Δ/tsl1Δ
CSM tps3Δ/tps3Δ tsl1Δ/tsl1Δ
Bb32 WT
Bb32 tps3Δ/tps3Δ
Bb32 tsl1Δ/tsl1Δ
Bb32 tps3Δ/tps3Δ tsl1Δ/tsl1Δ
SUPPLEMENTAL FIGURE 8. High-temperature growth (37°C) of tps3Δ, tsl1Δ, tps3Δ tsl1Δ mutants. The indicated strains were grown overnight in YNB + 2% glucose liquid at 30°C on a roller wheel before 10-fold serial dilutions were prepared in sterile water and spotted onto rich media (A) or minimal media (B) containing the indicated carbon sources (2% w/v for glucose and fructose; 3% v/v for glycerol; 2% v/v for ethanol). The cell concentration in the initial spotting dilution was OD600 = 1.0. Plates were incubated at 37°C for 2 days on rich media and 3 days on minimal media before photographing. Shown are representative images of three independent, biological replicates.

## Slide 18
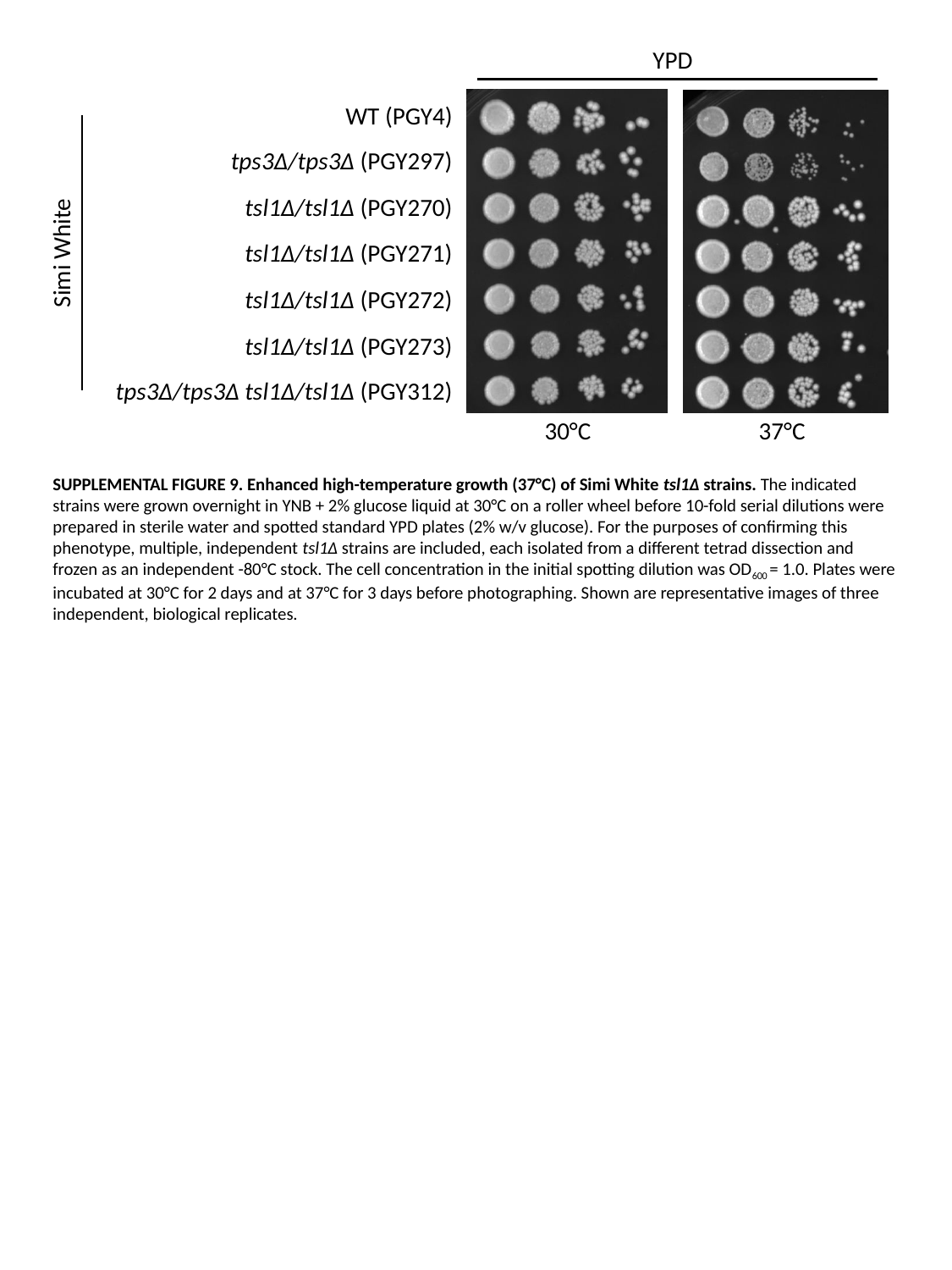

YPD
WT (PGY4)
tps3Δ/tps3Δ (PGY297)
tsl1Δ/tsl1Δ (PGY270)
tsl1Δ/tsl1Δ (PGY271)
Simi White
tsl1Δ/tsl1Δ (PGY272)
tsl1Δ/tsl1Δ (PGY273)
tps3Δ/tps3Δ tsl1Δ/tsl1Δ (PGY312)
30°C
37°C
SUPPLEMENTAL FIGURE 9. Enhanced high-temperature growth (37°C) of Simi White tsl1Δ strains. The indicated strains were grown overnight in YNB + 2% glucose liquid at 30°C on a roller wheel before 10-fold serial dilutions were prepared in sterile water and spotted standard YPD plates (2% w/v glucose). For the purposes of confirming this phenotype, multiple, independent tsl1Δ strains are included, each isolated from a different tetrad dissection and frozen as an independent -80°C stock. The cell concentration in the initial spotting dilution was OD600 = 1.0. Plates were incubated at 30°C for 2 days and at 37°C for 3 days before photographing. Shown are representative images of three independent, biological replicates.

## Slide 19
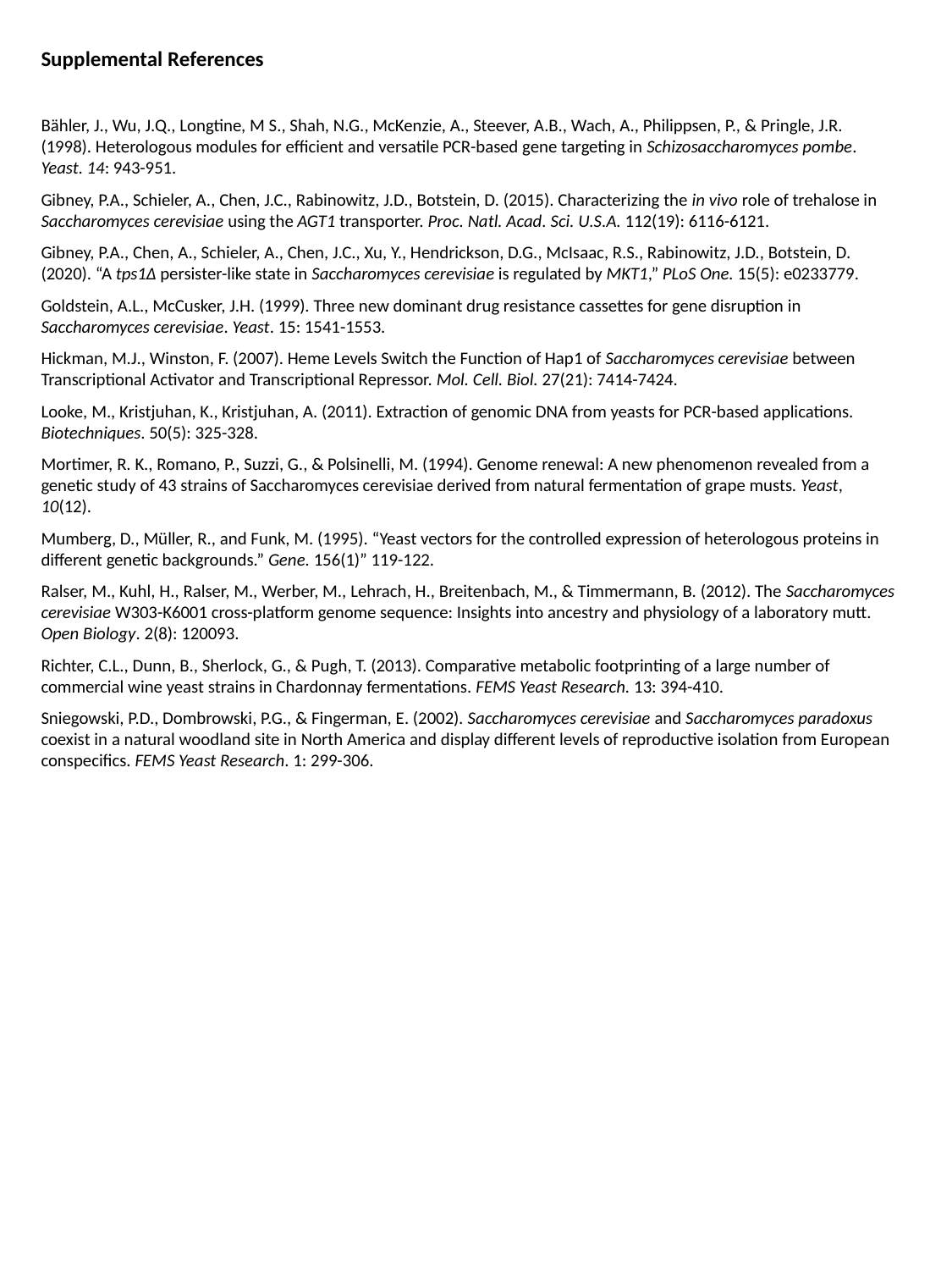

Supplemental References
Bähler, J., Wu, J.Q., Longtine, M S., Shah, N.G., McKenzie, A., Steever, A.B., Wach, A., Philippsen, P., & Pringle, J.R. (1998). Heterologous modules for efficient and versatile PCR-based gene targeting in Schizosaccharomyces pombe. Yeast. 14: 943-951.
Gibney, P.A., Schieler, A., Chen, J.C., Rabinowitz, J.D., Botstein, D. (2015). Characterizing the in vivo role of trehalose in Saccharomyces cerevisiae using the AGT1 transporter. Proc. Natl. Acad. Sci. U.S.A. 112(19): 6116-6121.
Gibney, P.A., Chen, A., Schieler, A., Chen, J.C., Xu, Y., Hendrickson, D.G., McIsaac, R.S., Rabinowitz, J.D., Botstein, D. (2020). “A tps1Δ persister-like state in Saccharomyces cerevisiae is regulated by MKT1,” PLoS One. 15(5): e0233779.
Goldstein, A.L., McCusker, J.H. (1999). Three new dominant drug resistance cassettes for gene disruption in Saccharomyces cerevisiae. Yeast. 15: 1541-1553.
Hickman, M.J., Winston, F. (2007). Heme Levels Switch the Function of Hap1 of Saccharomyces cerevisiae between Transcriptional Activator and Transcriptional Repressor. Mol. Cell. Biol. 27(21): 7414-7424.
Looke, M., Kristjuhan, K., Kristjuhan, A. (2011). Extraction of genomic DNA from yeasts for PCR-based applications. Biotechniques. 50(5): 325-328.
Mortimer, R. K., Romano, P., Suzzi, G., & Polsinelli, M. (1994). Genome renewal: A new phenomenon revealed from a genetic study of 43 strains of Saccharomyces cerevisiae derived from natural fermentation of grape musts. Yeast, 10(12).
Mumberg, D., Müller, R., and Funk, M. (1995). “Yeast vectors for the controlled expression of heterologous proteins in different genetic backgrounds.” Gene. 156(1)” 119-122.
Ralser, M., Kuhl, H., Ralser, M., Werber, M., Lehrach, H., Breitenbach, M., & Timmermann, B. (2012). The Saccharomyces cerevisiae W303-K6001 cross-platform genome sequence: Insights into ancestry and physiology of a laboratory mutt. Open Biology. 2(8): 120093.
Richter, C.L., Dunn, B., Sherlock, G., & Pugh, T. (2013). Comparative metabolic footprinting of a large number of commercial wine yeast strains in Chardonnay fermentations. FEMS Yeast Research. 13: 394-410.
Sniegowski, P.D., Dombrowski, P.G., & Fingerman, E. (2002). Saccharomyces cerevisiae and Saccharomyces paradoxus coexist in a natural woodland site in North America and display different levels of reproductive isolation from European conspecifics. FEMS Yeast Research. 1: 299-306.
